# Computational approaches to therapeutic antibody design: established methods and emerging trends

**DOI:** 10.1093/bib/bbz095

**Published:** 2019-10-18

**Authors:** Richard A Norman, Francesco Ambrosetti, Alexandre M J J Bonvin, Lucy J Colwell, Sebastian Kelm, Sandeep Kumar, Konrad Krawczyk

**Affiliations:** 1 Pistoia Alliance Inc., USA; 2 Sapienza University, Italy; 3 Utrecht University, Netherlands; 4 Cambridge University, UK; 5 UCB Pharma, UK; 6 Boehringer Ingelheim, USA; 7 NaturalAntibody, Germany

**Keywords:** homology modelling, therapeutic antibodies, docking, antibody–antigen complexes, databases

## Abstract

Antibodies are proteins that recognize the molecular surfaces of potentially noxious molecules to mount an adaptive immune response or, in the case of autoimmune diseases, molecules that are part of healthy cells and tissues. Due to their binding versatility, antibodies are currently the largest class of biotherapeutics, with five monoclonal antibodies ranked in the top 10 blockbuster drugs. Computational advances in protein modelling and design can have a tangible impact on antibody-based therapeutic development. Antibody-specific computational protocols currently benefit from an increasing volume of data provided by next generation sequencing and application to related drug modalities based on traditional antibodies, such as nanobodies. Here we present a structured overview of available databases, methods and emerging trends in computational antibody analysis and contextualize them towards the engineering of candidate antibody therapeutics.

## Introduction

Antibodies are immune system proteins that recognize the surfaces of foreign molecules (antigens) for subsequent elimination from the organism during an adaptive immune response [1] or self-antigens from healthy tissues in autoimmune diseases [2]. The antibodies have evolved to be versatile binders, capable of recognizing a wide variety of molecular surfaces [3]. Because of such favorable binding properties, antibodies have been harnessed for therapeutic purposes and are currently the largest class of biotherapeutics. Five of the current top-selling blockbusters are monoclonal antibodies: adalimumab and infliximab (anti-TNFα), rituximab (anti-CD20), bevacizumab (anti-VEGF), trastuzumab (anti-HER2/neu) and their market presence is still expanding [4].

Continued exploitation of antibodies for therapeutic purposes relies on more efficient ways to develop these molecules. Computational approaches hold promise in advancing the field by providing faster results than arduous experimental approaches that are the current standard in antibody discovery [5]. Established structural bioinformatics methods such as homology modelling [6, 7], protein–protein docking [8, 9] or protein interface prediction [10] are already used for rational antibody design [11–13]. There are also pharmaceutically focused computational approaches that aid in assessing the immunogenicity [14] and biophysical properties [15] of antibodies. The increasing number of structural [16], sequence [17] and experimental data [18–21] on antibodies being deposited in the public domain provides the necessary foundation for improving such data-driven methods. Specifically, the recent decade saw the advent of next generation sequencing (NGS) of B-cell receptor (antibody) repertoires [22]. NGS provides a snapshot of millions of antibody sequences sampled from the theoretically possible 10^12^–10^15^ antibody sequences in a human repertoire [23, 24]. Discerning the biases of antibody repertoires can provide insights into the natural diversity of the immune system [25]. Among others, such natural preferences can be used as a reference to assess the biophysical properties of therapeutic antibodies [13] or to develop naturally focused surface display libraries [26]. The accumulated methodology of computational antibody protocols could potentially be applied to novel antibody formats with intrinsically better biophysical properties, such as nanobodies [27]. Altogether, computational antibody analysis methods have matured enough to allow for wider applications in therapeutic development.

In this review we give a structured overview of the currently available databases, algorithms and resources for computational analysis and design of antibodies and the prediction of their binding mode to antigens. We provide context to these methods in the form of current efforts for therapeutic antibody design and delineate the emerging trends in the field.

## Antibody structure, function and therapeutic formats

**Figure 1:**
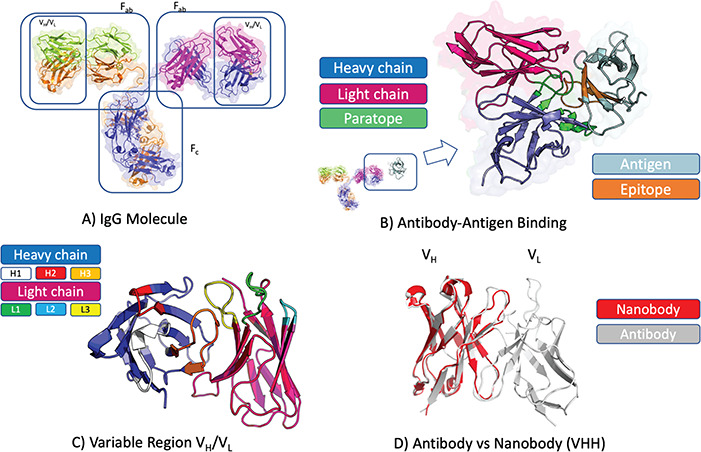
Antibody structure and binding. (**A**) Antibodies in soluble form often adopt the IgG isotype, a Y-shaped molecule consisting of two heavy chains (blue and amber) and two light chains (green and magenta). Each IgG molecule can be subdivided into an Fc and two Fab fragments through papain cleavage of the (hinge) region between these. At each end of a Fab fragment is a variable domain (VH/VL) involved in antigen binding. (**B**) Structure of an antibody VH(blue)/VL(magenta) in complex with cognate antigen (grey). The antibody paratope (light green) and antigen epitope (light brown) are highlighted. (**C**) Structure of an antibody VH(blue)/VL(magenta) highlighting the six hypervariable loops that make up the paratope; CDRH1 (white), CDRH2 (red), CDRH3 (amber), CDRL1 (green), CDRL2 (light blue), CDRL3 (yellow). (**D**) Comparison of antibody VH/VL domain (grey) and nanobody (red) structures. Nanobodies are devoid of the light chain, thus all the binding is mediated by the VH-homologous portion including its three CDR loops (CDRH1–3).

Antibodies, or Immunoglobulins (Ig), are produced in jawed vertebrates by B-cells. Each of the estimated 5 × 10^9^ B-cells in an organism [23] produces a distinct B-cell receptor (membrane-bound) or antibody (in soluble form) through somatic recombination of variable (V), diversity (D), joining (J) and constant (C) gene segments [28, 29]. The process of V(D) J recombination results in a Heavy (H) chain, assembled from V, D, J and C gene segments from the H chain locus, and a Light (L) chain, assembled from V, J and C gene segments from one of the L chain loci. In humans, the H and L chains can naturally assemble into five isotypes: IgG, IgD, IgE (all three monomers), IgA (dimer) and IgM (pentamer) [30]. The most biologically frequent format, IgG, consists of one crystallizable (F_c_) and two antigen binding (F_ab_) fragments and is illustrated in Figure 1A.

The F_ab_ contains the H and L chain variable segments (V_H_ and V_L_) that bind their cognate surface on the antigen, the epitope (Figure 1B). The V_H_ and V_L_ each harbor three hypervariable loops that define the complementarity determining regions (CDRs). The CDRs contain the majority of the antigen-binding residues, or paratope (Figure 1B–C). Upon antigen exposure, the antibody-producing B-cells undergo a natural process of affinity maturation, based on somatic hypermutation [31]. This process introduces mutations primarily in the CDR regions, which develop a specific and high-affinity binder. Together with the diversity introduced by V(D) J recombination, somatic hypermutation can produce an estimated 10^12^–10^15^ possible antibody sequences [23, 24]. The large number of diverse antibodies in an organism increases the probability of recognizing an arbitrary foreign antigen, thus initiating an immune response.

Despite the intrinsic sequence diversity in the CDRs, all hypervariable loops with the exception of CDRH3 adopt a constrained set of conformations termed canonical classes [32]. CDRH3 possesses the highest sequence and structural diversity of the six CDRs [33] and is very important for antigen recognition [34, 35]. Due to their central role in antigen recognition and binding, CDR loops undergo the most extensive engineering during development of monoclonal antibody (mAb) therapeutics [36, 37].

Standard mAb therapeutics have limited tissue penetration as a result of their large molecular weight (~150 kDa). As such, significant efforts, mostly based on modern protein engineering techniques, have been placed in the development of non-standard antibodies with superior properties. Some of these include F_ab_ domains and other modular formats with single chain Fvs (scFv) (linked V_H_ and V_L_ domains) as the main component, e.g. scFv, (scFv)2, diabody and minibody (reviewed by Holliger and Hudson [38], Farajnia *et al.* [39] and Kwon *et al.* [40]). In addition, bi-specific and polyspecific antibody formats, which can engage two or more antigens at the same time, have been developed, e.g. combining L and H chains from two different mAbs or fusing the V domains from two different mAbs to create an antibody with dual specificity. Bi- and tri-specific antibody formats aimed at solid tumors have been recently reviewed [41].

Single-domain antibody formats, called VHH or nanobodies, are found in camelids and sharks (Figure 1D). Single domain antibodies have attracted attention because of their smaller size and better biophysical properties with respect to antibodies (higher stability and lower suspected immunogenicity) [27]. Despite being half the size of a standard antibody variable domain (Figure 1D), nanobodies retain similar binding affinity and specificity as standard mAbs. Therefore, these molecules are of increasing therapeutic interest with the first nanobody therapeutic (caplacizumab) approved in 2018 [42].

## Antibody databases

Computational approaches to analyze and design antibodies rely on the availability of suitable datasets. Resources exist that curate the therapeutic antibody information, such as TABS (https://tabs.craic.com/) and SAbDab-Therapeutic-Antibodies [13]. Most other resources can be classified based on whether the content is sequence, structure or experimental information, with some databases being a combination of the three (Table 1). Most of these repositories collect both antibody and nanobody data; however, there are also some databases specializing only in the latter, e.g. sdAb-DB [58].

**Table 1:** Databases containing information on antibody and nanobody structure and sequence. Most of the databases are free for academic use. In cases where the authors made it clear that a commercial version is available, this is indicated next to the database name. In some cases, such as IMGT or SKEMPI, conditions for non-commercial reuse are defined. In such cases, the authors of the respective databases should be contacted for details on commercial re-use of their material. Example contents of the databases are summarized in Supplementary Section 1. An up-to date list of antibody-related database resources is maintained at http://naturalantibody.com/tools

Database Name	Link	Description	Reference
TABS (commercial use)	https://tabs.craic.com/users/sign_in	Database of therapeutic antibodies	n/a
SAbDab-therapeutic antibodies	http://opig.stats.ox.ac.uk/webapps/sabdab-sabpred/Therapeutic.html	Database of therapeutic antibodies linked to structures	[13]
Andrew Martin’s Antibody Resources	http://www.bioinf.org.uk/abs/	Resources on antibody-related analytics	n/a
AAAAA	https://www.bioc.uzh.ch/plueckthun/antibody/index.html	Resources on antibody-related analytics	[43]
AbMiner	https://discover.nci.nih.gov/abminer/	Database of available monoclonal antibodies	[44]
IgPdb	http://cgi.cse.unsw.edu.au/~ihmmune/ IgPdb/information.php	Database of inferred allelic variants for immunoglobulins	[45]
IMGT®	http://www.imgt.org/	Leading antibody genetics database	[46]
Abysis (commercial license available)	http://www.abysis.org/	Sequence and structural data on antibodies	[47]
DIGIT	http://circe.med.uniroma1.it/digit/help.php	Antibody sequence database	[48]
Ireceptor	http://ireceptor.irmacs.sfu.ca/	NGS sequence data on B-cell receptors	[49]
Observed Antibody Space	http://antibodymap.org/oas	NGS sequence data on B-cell receptors/antibodies	[17]
SystimsDB (commercial license available)	https://www.systimsdb.ethz.ch	NGS sequence data on B-cell and T-cell receptors	n/a
PCLICK	http://mspc.bii.a-star.edu.sg/minhn/cluster_pclick.html	Clusters of antibody–antigen interactions	[50]
PyIgClassify (commercial license available)	http://dunbrack2.fccc.edu/PyIgClassify/	Database of CDR canonical classes	[51]
Structural Antibody Database	http://opig.stats.ox.ac.uk/webapps/sabdab-sabpred/Welcome.php	Self-updatable database of antibody/nanobody structures	[16]
AbDb	http://www.bioinf.org.uk/abs/abdb/	Database of antibody structures	[52]
Immune Epitope Database	http://iedb.org	Manually curated epitope data	[18]
AntigenDB	http://crdd.osdd.net/raghava/antigendb/	Antigen database	[53]
PDBBind	http://www.pdbbind.org.cn/	Affinity data on proteins in the PDB	[54]
Ab-Bind	https://github.com/sarahsirin/AB-Bind-Database	Mutational antibody data related to binding affinities	[19]
SKEMPI (non-commercial use)	https://life.bsc.es/pid/skempi2/	Not-antibody specific interaction database	[55, 56]
Non-redundant Nanobody database	https://www.sciencedirect.com/science/ article/pii/S2352340919301052	Non-redundant structures of nanobodies	[57]
Single Domain Antibody Database	http://sdab-db.ca/	Sequence and structural data on nanobodies	[58]
Institute for analysis and collection of nanobodies	http://ican.ils.seu.edu.cn/	Sequences and structural data on nanobodies	[59]

### Sequence databases

The leading database of germline antibody sequences is the International Immunogenetics Information System (IMGT) [46] and it is widely used to derive gene assignments for recombined antibodies. Most other resources typically store the recombined sequences of the variable regions (V_H_ and V_L_). Such databases can be divided into those that specialize in single sequence depositions, e.g. DIGIT [48], Abysis [47] or bulk raw reads produced by NGS experiments, e.g. iReceptor [49], Observed Antibody Space [17]. Tools such as DIGIT and Abysis source their data from the European Nucleotide Archive (ENA) [60] and the National Center for Biotechnology Information (NCBI) [61]; their sequence volumes are of the order of 10^5^ and contain multiple artificially engineered sequences. These data typically originate from single molecule depositions, often derived by Sanger sequencing, and thus can be regarded as of high quality. Repositories containing NGS data derive their contents from the raw sequence reads deposited in multiple repositories, including ENA and NCBI. Due to the high-throughput nature of NGS, sequence volumes are of the order of 10^8^ and are associated with non-trivial error rates [62]. The raw sequences are annotated with antibody-specific information such as CDRs, numbering schemes and wherever available experimental data on the immune state of the donor at the point of sequence collection. Certain resources, such as Observed Antibody Space, address this by offering an annotation of the predicted sequence errors [62]. The NGS databases typically offer only the unpaired heavy and light chains; however, as the paired NGS technology becomes more mainstream, it is to be expected that such data will also become publicly available [63, 64].

### Structure databases

The Protein Data Bank (PDB) is the main global repository of 3D structure information for proteins [65]. Resources that mine the PDB for antibody fragments such as canonical classes (PyIgClassify [51]), antibody–antigen interaction data (PCLICK [50]) or their entire structures (IMGT/3D-Structure-DB [66], Structural Antibody Database (SabDAb [16]), Abysis [47] and AbDb [52]) exist. According to SAbDab, of the approximately 150 000 structures deposited in the PDB to date as many as 3500 are identified as containing at least one antibody (or nanobody) chain. SAbDab specifically allows for a bulk download of its weekly updatable database, providing an up-to-date resource for applications such as antibody modelling or docking. SabDab and Abysis allow retrieval of particular structures given a query antibody sequence (SAbDab) or by using more advanced features such as canonical classes of the CDRs (Abysis). Other resources such as the Immune Epitope Database (IEDB) [18] link structural information to experimentally derived epitope data.

### Experimental databases

Sequence and structure data can be further enriched with antibody-specific experimental information. Data on epitopes targeted by antibodies can be readily downloaded from the IEDB that now links such information to epitope-specific antibody sequences [67]. One of the crucial pieces of information to characterize antibody–epitope interactions is the binding affinity. Such data is contained in resources such as SAbDab and PDBBind [54]. Other, more specialized, resources exist such as Ab-Bind [19], which hold data on 1101 mutations across 32 antibody complexes, and SKEMPI [55], which curates binding energy data for available structures but is not limited to antibody information only.

## Computational characterization of antibodies

The increasing availability of antibody-specific sequence, structure and experimental data allows development of bioinformatics tools facilitating antibody engineering (Table 2). Routine bioinformatics methods such as homology modelling and protein–protein docking can be harnessed to guide the engineering of therapeutic antibodies [5]. Antibody-based therapeutics are developed via well-established processes that can be broadly categorized into Lead Identification and Lead Optimization. During Lead Identification animal immunization or surface display technologies are used to generate a large number of ‘hit’ molecules, which need to be further triaged. Following various rounds of further screening and design during Lead Optimization, a small number of high affinity lead candidates are selected. During Lead Identification and Optimization, molecules are assessed for unfavorable characteristics such as immunogenicity or poor biophysical properties. This assessment of ‘developability’ risk is of key importance before undergoing clinical trials and to ensure the successful development of a lead candidate into a stable, manufacturable, safe and efficacious therapeutic. Computational methods, such as homology modelling, docking or interface prediction can be used during the Lead Identification and Optimization phases to generate 3D models of the antibodies and predict or identify the key residues involved in antigen binding.

**Table 2:** Computational antibody tools. The algorithms or software packages were grouped by the core area of their application: Antibody Annotation/Numbering, Structural Antibody Modelling, Antibody–Antigen Interface Prediction, Antibody Design and Pharmaceutically-specific applications. We provide the name for each method together with a reference and weblink to the software if available. Some resources are currently not maintained, in which case we suggest contacting the authors directly. A web-based version of this table where the resources listed below and newly released ones are curated is maintained at http://natuarlantibody.com/tools

A. Antibody Annotation/Numbering	Role	Link	Reference
IgBLAST	Raw data processing	https://www.ncbi.nlm.nih.gov/igblast/	[68]
IMGT V-Quest	Raw data processing	http://www.imgt.org/IMGTindex/V-QUEST.php	[69]
MiXCR	Raw data processing	https://mixcr.readthedocs.io/en/master/	[70]
Immcantation	Raw data processing	https://immcantation.readthedocs.io	[71, 72]
IgRec	Raw data processing	https://yana-safonova.github.io/ig_repertoire_constructor/	[73]
ImmuneDiversity	Raw data processing	https://bitbucket.org/ImmunediveRsity/immunediversity/	[74]
IMSEQ	Raw data processing	http://www.imtools.org/	[75]
Partis	Raw data processing	https://github.com/psathyrella/partis	[76]
IGoR	Raw data processing	https://github.com/qmarcou/IGoR	[77]
Vidjil	Raw data processing	http://www.vidjil.org/	[78, 79]
ImmuneDB	Raw data processing	https://immunedb.readthedocs.io/en/latest/	[80]
AbRSA	Numbering	http://cao.labshare.cn/AbRSA/	[81]
Abnum	Numbering	http://www.bioinf.org.uk/abs/abnum/	[82]
ANARCI	Numbering	http://opig.stats.ox.ac.uk/webapps/sabdab-sabpred/ANARCI.php	[83]
B. Structural Antibody Modelling	Role	Link	Reference
AbodyBuilder	Full FV modelling	http://opig.stats.ox.ac.uk/webapps/sabdab-sabpred/Modelling.php	[84]
LYRA	Full FV modelling	http://www.cbs.dtu.dk/services/LYRA/index.php	[85]
PIGS	Full FV modelling	https://cassandra.med.uniroma1.it/pigspro/	[86]
Kotai Antibody Builder	Full FV modelling	http://kotaiab.org/	[87]
RosettaAntibody	Full FV modelling	http://rosie.rosettacommons.org/antibody	[88, 89]
BIOVIA	Full FV modelling	https://www.3dsbiovia.com/	[90]
MoFvAb	Full Fv Modelling	-	[91]
WAM	Full Fv Modelling	-	[92]
BioLuminate	Full Fv Modelling	https://www.schrodinger.com/products/bioluminate	[93]
MOE	Full Fv Modelling	https://www.chemcomp.com/	[94]
ABGEN	Full Fv Modelling	-	[95]
AbPredict	Full FV modelling	http://abpredict.weizmann.ac.il/bin/steps	[96]
SmrtMolAntibody	Full FV modelling	https://www.macromoltek.com/	[97]
PEARS	Ab-specific side chain prediction	http://opig.stats.ox.ac.uk/webapps/sabdab-sabpred/PEARS.php	[98]
H3LoopPred	Antibody specific loop prediction	-	[99]
SCWRL	Side Chain Prediction	http://dunbrack.fccc.edu/scwrl4/	[100]
BetaSCPWeb	Side Chain Prediction	http://voronoi.hanyang.ac.kr/betascpweb/	[101]
SPHINX	Antibody specific *ab initio* loop prediction	http://opig.stats.ox.ac.uk/webapps/sabdab-sabpred/Sphinx.php	[102]
FREAD	Database-search loop prediction	http://opig.stats.ox.ac.uk/webapps/fread/php/	[103]
PLOP	*Ab initio* loop prediction	http://www.jacobsonlab.org/plop_manual/plop_overview.htm	[104]
Chothia Canonical Assignment	CDR Canonical structure prediction	http://www.bioinf.org.uk/abs/chothia.html	Based on [105]
SCALOP	CDR Canonical structure prediction	http://opig.stats.ox.ac.uk/webapps/sabdab-sabpred/SCALOP.php	[106]
Roche V_H_/V_L_ orientation	VH/VL orientation	-	[107]
Rosetta V_H_/V_L_ orientation	VH/VL orientation	Rosetta Suite	[108]
AbAngle	VH/VL orientation	http://opig.stats.ox.ac.uk/webapps/abangle/index.html	[109]
Antibody i-Patch	Paratope Prediction	http://opig.stats.ox.ac.uk/webapps/sabdab-sabpred/ABipatch.php	[110]
Paratome	Paratope Prediction	http://ofranservices.biu.ac.il/site/services/paratome/	[111]
ProABC	Paratope Prediction	http://circe.med.uniroma1.it/proABC/	[112]
Parapred	Paratope Prediction	https://github.com/eliberis/parapred	[113]
AntibodyInterface Prediction	Paratope Prediction	https://github.com/sebastiandaberdaku/AntibodyInterfacePrediction	[114]
AG-FAST-Parapred	Paratope Prediction	-	[115]
ISMBLab-PPI	Protein contact prediction, applied to paratopes	http://ismblab.genomics.sinica.edu.tw/predict.php?pred=PPI	[3]
Rapberger *et al.* 2007	Ab-specific epitope prediction	-	[116]
PEASE	Ab-specific epitope prediction	http://ofranservices.biu.ac.il/site/services/epitope/index.html	[117, 118]
EpiPred	Ab-specific Epitope Prediction	http://opig.stats.ox.ac.uk/webapps/sabdab-sabpred/EpiPred.php	[119]
Jespersen *et al.*	Ab-specific Epitope Prediction	-	[120]
EpiScope	Ab-specific Epitope Prediction	-	[121]
MabTope	Ab-specific Epitope Prediction	-	[122]
ASEP	Ab-specific Epitope Prediction	-	[123]
BEPAR	Ab-specific Epitope Prediction	-	[124]
ABEPAR	Ab-specific Epitope Prediction	-	[125]
ClusPro	Ab-specific docking	https://cluspro.bu.edu/login.php	[8, 126]
surFit	Ab-specific docking	https://sysimm.ifrec.osaka-u.ac.jp/docking/main/	[127]
SnugDock	Ab-specific docking	http://rosie.graylab.jhu.edu/snug_dock	[9, 89]
FRODOCK	Ab-specific docking	http://frodock.chaconlab.org/	[128]
DockSorter (ab-specific scoring)	Ab-specific docking scoring	http://www.stats.ox.ac.uk/~krawczyk/dockingsupp.html	[110]
Hex	Docking, not antibody specific	http://hex.loria.fr/	[129]
ZDOCK	Docking, not antibody specific.	http://zdock.umassmed.edu/	[130]
HADDOCK	Docking, not antibody specific	https://haddock.science.uu.nl/services/HADDOCK2.2/	[131, 132]
ATTRACT	Docking, not antibody specific	http://www.attract.ph.tum.de/services/ATTRACT/attract.html	[133]
GRAMM-X	Docking, not antibody specific	http://vakser.compbio.ku.edu/resources/gramm/grammx/	[134]
pyDockWeb (pyDock, FTDock)	Docking, not antibody specific	https://life.bsc.es/pid/pydockweb	[135]
Swarmdock	Docking, not antibody specific	https://bmm.crick.ac.uk/~svc-bmm-swarmdock/	[136]
PatchDock	Docking, not antibody specific	https://bioinfo3d.cs.tau.ac.il/PatchDock/	[137, 138]
D. Antibody Design	Role	Link	Reference
OPTCDR	Design Protocol	http://www.maranasgroup.com/submission/OptCDR_2.htm	[139]
OPTMaven	Design Protocol	https://github.com/maranasgroup/OptMAVEn_2.0	[140, 141]
RosettaAntibodyDesign	Design Protocol	https://www.rosettacommons.org/docs/latest/application_documentation/antibody/RosettaAntibodyDesign	[142]
AbDesign	Design Protocol	https://www.rosettacommons.org/node/9206	[12, 143]
Humanness Score	Humanization	http://www.bioinf.org.uk/abs/shab/	[14]
Humanizer	Humanization	https://drive.google.com/file/d/1seCQYMlMG4_oC1-0EjiDhZHnMf9D-1R5/view?usp=sharing	[141]
Tabhu	Humanization	http://circe.med.uniroma1.it/tabhu/	[144]
Human String Content	Humanization	-	[145]
Human String Content	Humanization	-	[145]
T20 Score	Humanization	https://dm.lakepharma.com/bioinformatics/	[146]
CODah	Humanization	-	[147]
Developability Index	Developability	-	[148]
Delayed HIC retention prediction	Developability	-	[149]
Therapeutic Antibody Profiler	Developability	http://opig.stats.ox.ac.uk/webapps/sabdab-sabpred/TAP.php	[13]
Lonza	Developability	-	[15]

### Antibody numbering

The first step in antibody computational analysis is to map the antibody sequences onto a standardized reference framework (Table 2A). Raw nucleotide sequences of variable regions can be translated into amino acids by aligning them to germline sequences, thus identifying the V, D and J regions. This can be achieved by programs such as IgBLAST [68] or IMGT V-Quest [69] and multiple other tools aimed at processing raw antibody data (Table 2A, reviewed in [150]). Similarities between antibody amino acid sequences further allow for the creation of a standardized reference framework, or numbering scheme, giving each variable region amino acid an identifier [151]. The numbering schemes contextualize each position within the structure of an antibody, allowing for rapid delineation of CDR and framework regions. Since the seminal work to define a standard numbering scheme for antibodies was carried out by Kabat in 1970 [152], the Chothia [32] and IMGT [153] schemes have been adopted as the main alternatives. Additional numbering schemes such as Contact [154], North [155], WolfGuy [107] and Aho [43] exist but these are less prevalent. Kabat and IMGT definitions are based on sequence alignments identifying conserved positions in the variable region [152, 153] whereas Chothia takes into account the 3D structure of the CDR loops. The antibody numbering scheme developed by the Chemical Computing Group (CCG) combines several antibody numbering schemes and offers a broader definition of CDR boundaries based on Martin and collaborators’ CDR definitions (http://www.bioinf.org.uk/abs/#martinnum). To the best of our knowledge, there are three freely available software packages to perform numbering of antibodies, ANARCI [83], Abnum [82] and AbRSA[81], to act as the first step in computational antibody analysis such as homology modelling.

### Antibody modelling

Structural antibody modelling creates a 3D structure from its sequence alone, based on existing knowledge of antibody structures in particular and protein structures in general. The high degree of antibody sequence and structure conservation in the framework region and the five canonical loops leads to an overall high accuracy of antibody homology modelling [7].

**Figure 2:**
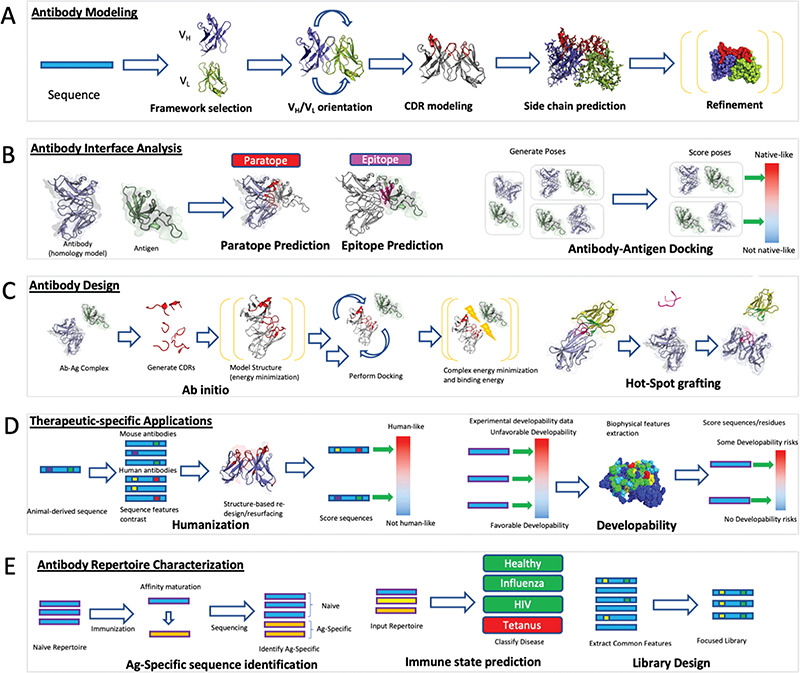
Computational antibody methods schematic. (A) Antibody modelling produces three dimensional coordinates from the sequence of an antibody. Framework templates are identified and the VH/VL domains can be oriented with respect to each other if the two regions originate from different molecules. CDRs are modelled onto the framework followed by side-chain prediction and refinement of the entire structure by energy minimization. (B) Antibody interface prediction identifies the residues on the antibody (paratope) that are in contact with the antigen (epitope). This is a special case of molecular docking in which the antibody–antigen docking aims to recapitulate the complex between the antibody and the antigen. (C) Antibody design optimizes the binding of an antibody against an epitope of choice through a series of modelling, docking and energy minimization steps. In *ab initio* design, novel paratopes are generated computationally and their structural stability and binding propensity against the cognate epitope assessed by energy functions. Hotspot grafting involves transferring known interaction motifs from the antigen partner protein to an antibody template. (D) Antibodies need to be immunologically safe and have favorable biophysical properties in order to be administered to humans. Humanization involves modifying an animal-derived sequence to resemble one with a higher degree of human amino acid content without affecting its affinity and specificity. Developability-specific applications annotate regions on the surface that might lead to poor solubility or aggregation altogether. (E) Entire antibody repertoires can be used to draw information on the mechanics of the adaptive immune system. Identification of antigen-specific sequences post-vaccination can identify antibodies that could bestow passive immunity. The dynamic state of the repertoire can be analyzed to identify diseases in the organism. The diversity of antibodies can be harnessed to create surface display libraries recapitulating naturally evolved preferences and advantages.

Antibody modelling generally follows a five-step process (Figure 2A). The first step is selection of a suitable framework template that can harbor the CDR loops. This is typically achieved by finding close sequence matches to the H and L chains in available databases [16]. The second step involves accurate determination of the relative orientation of the V_H_ and V_L_ domains, which is crucial to determine the correct shape of the paratope [107, 109]. Specific algorithms have been developed for this and incorporated into available software packages such as AbAngle [109]. The third step involves modelling of the CDR loops. Knowledge-based methods are currently capable of providing accurate predictions for the five canonical loops, but CDRH3 remains a challenge [156]. Antibody-specific knowledge-based approaches are fast and accurate if a template is available [103]. If there is no suitable template, as can be often the case with CDRH3, more computationally expensive *ab initio* approaches can be employed that generate a large set of novel loops. The biggest challenge in such *ab initio* modelling remains selection of best loop models among those generated [102]. Hybrid methods such as Sphinx [102] combine knowledge-based and *ab initio* approaches to provide better all-round predictions irrespective of the presence or absence of a priori structural information. The fourth step involves building and refining of the side-chains [98]. Here, protein-generic approaches such as SCWRL [100] can be employed although it has been demonstrated that an antibody-focused approach, such as PEARS, could yield better results [98]. The final antibody model can be further refined by optimizing the energetic packing of the molecule, through packages such as Rosetta [89].

Available tools that employ the methods described above are summarized in Table 2B, including those specific for individual steps in the modelling process. The modelling protocols are currently available via free-to-use web-servers, e.g. PIGS [86], AbodyBuilder [84]; as commercial packages, e.g. Biovia from Accelrys (https://www.3dsbiovia.com/), SmrtMolAntibody from Macromoltek (https://www.macromoltek.com/), MOE from CCG (https://www.chemcomp.com/) and BioLuminate from Schrodinger Inc. (https://www.schrodinger.com/products/bioluminate); or for local installation, e.g. AbPredict [96], Rosetta [89]. The tools vary radically in run-times, with tools such as AbodyBuilder capable of producing a model in around 60 s, to Rosetta-based frameworks that can take up to several hours. Despite different run times, the tools produce comparable results as exemplified by the Antibody Modelling Assessment II [7], a benchmarking experiment in which blinded predictions using some of the aforementioned tools were conducted. AMA II reported that the overall accuracy of modelling the entire antibody F_V_ is 1.1 Å Root Mean Square Deviation (RMSD) on average, with the most challenging region being the CDRH3, which is modelled to >5 Å RMSD in some targets. Such results cannot rival the accuracy of experimentally derived structures, but a model with 1.0 Å RMSD, especially across the CDR region, can be used as a rapid proxy to delineate structural features of the molecule. Modelled structures can be used at the Lead Identification stage to select surface exposed paratope residues for mutations [110] or to characterize the binding with respect to the cognate epitope [119]. Accurate structural information can be used during the Lead Optimization stage to assess various developability indicators, such as hydrophobicity [13] that rely on accurate models of the molecular surface of the paratope and epitope.

### Interface prediction and antibody–antigen docking

Understanding the epitope–paratope interactions at the atomic level is key to rational development of effective therapeutics. The ‘gold standard’ for obtaining this information is by experimentally determining the 3D structure of the antibody–antigen complex using X-ray crystallography. Other structural methods such as cryo-electron microscopy (cryoEM) or nuclear magnetic resonance (NMR) can be used but the size of the complexes makes it challenging for the latter. Experimental methods can be very time and resource consuming with success not being guaranteed. Thus, computational methods that predict antibody–antigen contact surfaces could be a rapid alternative during therapeutic discovery efforts. These methods can be categorized into those that predict the paratope, the epitope or the entire antibody–antigen complex (Figure 2B and Table 2C).

About half of the 40–50 residues in the CDRs are in direct contact with the antigen, forming the paratope [157–159]. Analyses of high resolution crystal structures of antigen-antibody complexes show that the framework residues can bury a substantial amount of surface area upon complex formation [159, 160]. Computational predictors of paratopes address this problem (Table 2C) and they could have an impact in constraining and guiding mutational choices for rational affinity engineering of therapeutics during Lead Optimization. They can also provide valuable information to guide the modelling of antibody–antigen complexes during Lead Identification. For instance, statistical approaches such as Antibody i-Patch [110] assign a score to each residue with respect to its propensity to be part of the paratope, with high-scoring residues offering potential candidates for mutagenesis. Since not all paratope residues are constrained to the CDRs, tools such as Paratome [111, 159] can be used to identify positions in the framework region that might contribute to antigen recognition as well. Recently, Antibody i-Patch and Paratome were outperformed by machine learning approaches such as the random forest-based proABC [112], support-vector machine-based AntibodyInterfacePrediction [114] and the deep learning-based, Parapred [113] and AG-Fast-Parapred [115]. To the best of our knowledge AntibodyInterfacePrediction and AG-Fast-Parapred are currently the best performing paratope prediction methods as compared to previously available methods; however, they were not compared against one another. AG-Fast-Parapred predictions are obtained with reference to the antigen; therefore, as the authors suggest, the method might be applicable to epitope prediction as well.

Accurate delineation of an epitope is an important step in characterizing the function of an antibody [161] (Figure 2B). From a therapeutic perspective, knowledge of the epitope can be used for rational design in targeting an immunogenic region for vaccine development [162]. From a legal perspective, characterization of the antibody–antigen interaction is of importance when filing therapeutic antibody patents [163]. To achieve such goals, epitopes can be identified by various experimental methods [163] or be predicted by computational protocols [164]. Methods for computational epitope prediction can be divided into predictors of linear epitopes, which focus on identifying contiguous stretches of primary amino acid sequence, and conformational epitope predictors, which aim to identify the 3D configuration of the epitope. The majority of epitopes are conformational in nature therefore predictors that use structural antigen information offer more accurate results than linear methods [165, 166]. Many epitope prediction methods do not include information on the antibody, thus focusing on identifying generic immunogenic molecular surfaces [167]. However, arbitrary molecular surfaces appear to be indistinguishable from epitope regions [167–169] suggesting that predictions should be performed with reference to a particular antibody [116, 118–120]. We summarize the linear and conformational epitope predictors in Supplementary Information and those following the new paradigm of including antibody in providing epitope predictions (antibody-specific predictors) in Table 2C. Antibody-specific epitope prediction was first addressed by Rapberger and co-workers in 2007 [116] and subsequently by methods such as ASEP [123], BEPAR [124], ABEpar [125], EpiPred [119], PEASE [117, 118], MabTope [122] and Jespersen *et al.* [120]. The most recent approaches, such as those by MabTope and Jespersen *et al.*, perform antibody-specific epitope predictions in conjunction with protein-protein docking to offer information on the paratope-epitope pairings.

Paratope and epitope prediction can offer useful information on antibody–antigen recognition, which can be exploited for therapeutic design but these methods do not provide information about the specific interactions involved in antibody–antigen binding. This issue is addressed by antibody–antigen docking, a specialized application of the broader field of molecular docking [170] (Figure 2B). Molecular docking aims to predict the biological complex starting from the unbound proteins. It typically involves two steps; the sampling step, during which thousands of possible complex conformations are generated (e.g. antibody-specific ClusPro [8, 126], SnugDock [9, 89] and general protein HADDOCK [131, 132], ZDOCK [130]) and the scoring step, where the conformations are ranked according to a specific scoring function (e.g. antibody-specific DockSorter [110] and general protein ZRANK [171], FireDock [172], SIPPER[173]) to discriminate models that are closer to the native conformation. According to the sampling strategy used during the simulation, docking methods can be classified into two categories. The first class includes algorithms that perform a global search around the whole interfaces of the components without taking into account previous information about the binding region (*ab initio* docking). On the other hand, experimental or predicted information about the binding interface is often available and can be used to drive the sampling during docking (information-driven, local or integrative docking) [174]. Both classes can benefit from available information during the scoring step to select models that are consistent with the available information about the interaction. Additionally, inputs from experimental studies such as hydrogen-deuterium exchange (HDX) coupled with mass spectrometry and mutational analyses can help refine the computational models of antibody–antigen complexes [175, 176].

Another important aspect to be considered in the study of the biomolecular interactions regards the conformational changes that the molecules undergo upon binding. Most docking algorithms do not take into account conformational changes of the components, performing only ‘rigid-body docking’*.* Examples of widely used rigid-body docking software are ClusPro [8, 126], ZDOCK [130] and PatchDock [137, 138]. Since in most cases flexibility of the molecule is a crucial factor to be considered [177], approaches that tackle this problem have been developed over the years. Examples of such methods are for example Swarmdock [136], HADDOCK [132] and SnugDock [9].

All of the aforementioned methods allow the user to provide information about the binding interface using different strategies to implement the methodologies during the simulation. This feature is particularly relevant in the case of antibody–antigen docking as CDRs in particular offer a reasonable proxy of the binding interface. In fact, some docking methods such as ClusPro and PatchDock are able to automatically define the antibody CDRs in order to use this information during the docking process. The most challenging aspect is identification of the epitope since, despite the great efforts of the community in developing accurate epitope prediction methods, existing systems still do not provide reliable predictions, limiting their applicability in molecular docking.

HADDOCK is one of the few methods that can encode a variety of experimental and predicted information into restraints throughout the entire docking process to both drive and score the generated models following a data-driven strategy. Restraints can be derived from various experimental sources such as NMR chemical shifts perturbations, HDX and chemical cross-linking detected by mass spectrometry and mutagenesis data. In the case of antibodies, it has recently been demonstrated that HADDOCK is able to already provide high quality models when only a loose definition of the epitope and the hypervariable loops of antibodies are used to drive the docking [178]. Despite the availability of experimental data and their use to drive the docking and/or score the generated models, accurate prediction of biomolecular complexes remains a real challenge with much room for improvement. Current docking methods still cannot rival the reliability of X-ray crystallography-derived structures and their performance is regularly assessed by the Critical Assessment of Predicted Interactions (CAPRI) [179]. Here, scientists are typically provided sequences of the interacting partners (or in rare cases the structures of the unbound components) and are tasked with predicting the native complex. CAPRI rounds over the years have catalyzed and demonstrated improvements in protein–protein docking methodology. Targets consisting of antibody–antigen complexes are regularly included. Therefore, as methods for antibody–antigen interaction prediction improve, it is to be expected that triangulation of results from paratope prediction, epitope prediction and antibody–antigen docking methods could provide a relatively fast and cost-effective route to obtaining reliable information on which to base rational antibody design decisions.

## Computational methods for therapeutic antibody discovery

### Antibody design

Antibody modelling and interface prediction/analysis tools can be used to create novel molecules *ab initio* during Lead Identification or as auxiliary tools during Lead Optimization (Figure 2C and Table 2D). The availability of an antigen structure opens up the possibility to develop a novel antibody binder computationally [180]. The seminal work on the subject was published by Lippow and co-workers, who computationally improved the binding of an antibody against its target, starting from an existing structural complex [181]. The authors performed comprehensive computational mutagenesis of the CDRs and assessed the binding of the novel designs using the CHARMM energy function [182]. Selected molecules had better affinity for the target, demonstrating that in some scenarios computational approaches alone can be used for affinity maturation.

Since then, four methods have been made available: OptCDR [139], OptMAVEn[140], AbDesign [143] and RosettaAntibodyDesign [142]. These protocols can be broadly categorized as *ab initio* since they aim to design novel paratopes through four sequential steps: CDR generation, modelling, antibody–antigen docking and binding energy evaluation. OptCDR and RosettaAntibodyDesign generate CDR conformations by sampling known canonical classes and modelling the CDRH3 loop. In contrast, OptMAVEn and AbDesign generate molecules by modular design in a process akin to that of V(D) J recombination. The new CDRs are grafted onto a framework and the structures are energy-minimized by well-established energy functions such as RosettaEnergy [183] or CHARMM [182]. The affinity of each variant is further optimized by docking the antibody onto the target antigen and scored by assessing the interaction energy between antibody and antigen. *Ab inito* methods such as these are still emerging and although some of them demonstrated the validity of their constructs experimentally there exists for them to be validated across multiple projects in industrial setting to assess their utility.

The four methods outlined above facilitate the re-design of CDRs to improve antibody stability and affinity through a combination of conformational and free energy change optimization upon modification of specific residues. In contrast, Liu and co-workers validated an approach in which binding site motifs from existing protein–protein complexes were transferred directly onto an antibody in a process termed ‘hot-spot grafting’ [11]. A further approach to data mining existing structures to improve antibody affinity is ‘re-epitoping’, pioneered by Ofran and collaborators [184]. Here, existing antibodies are tested for complementarity to a target epitope and the best candidates are used to computationally construct focused surface display libraries. This protocol is exemplary in showing how the computational constructs can guide the traditional discovery methods to accelerate the discovery of therapeutic lead candidates.

The methods outlined above offer the potential for discovering specific and selective binders computationally, reducing the experimental effort during the Lead Identification stage. Such binders need to be further developed during Lead Optimization stage by assessing their immunogenicity and overall ‘developability’ potential through understanding of their biophysical properties.

### Immunogenicity prediction

A large proportion of currently developed antibodies are discovered by animal immunizations. Molecules raised in animals, such as mice, carry the risk of inducing an immunological response in humans in the form of anti-drug antibodies (ADAs). To avoid such issues, animal-derived antibodies undergo a process called humanization [185, 186]. During this process the CDRs from the (typically) mice-derived antibodies are grafted onto human frameworks, or alternatively, the mice-derived frameworks are engineered to resemble human ones. Traditionally, humanization involves comparing the animal-derived sequence with approximately 1000 human germline sequences before selecting the appropriate template. Germline sequences however only offer a limited view of overall mutational antibody diversity, which can be addressed by computational humanization, comparing the animal-derived therapeutic to the distribution of amino acids in human antibody sequences (Figure 2D, Table 2E).

This was addressed by Tabhu [144], a web-server that compares a query therapeutic sequence to thousands of recombined variable region sequences from DIGIT [48] and serves as a reference in humanization. Although this takes into account antibody sequence diversity, humanization is a complex process where simple pairwise homology and alignments might be insufficient. Thus, statistical approaches to assess the ‘humanness’ of the query sequence have been developed (Figure 2D and Table 2E). One of the earliest examples is the Humanness score by Andrew Martin’s group [14] in which the authors contrasted the distribution of amino acids in antibody sequences in humans and mice. This allowed them to develop a statistical score indicating whether a query sequence is close in its amino acid content to the human distribution and provided a global metric based on the entire antibody variable region. Since immunogenicity is mediated by short peptides on the molecule, Lazar and co-workers developed the Human String Content (HSC) score that takes into account short (9-mer) sequences along the variable region to indicate regions requiring modification to conform with the human amino acid distribution [145]. Humanness score and HSC are primarily sequence distance-based approaches and it has been demonstrated that more sophisticated methods that take the positional correlations between residues into account could be superior [187, 188]. These methods remain sequence-based and do not explicitly use the structure of the antibody to be humanized, although HSC uses a contact-based score derived a priori. Since the immunogenic portions of the antibody would naturally be found on the surface, structural modelling can readily identify solvent-exposed positions aiding in a process called re-surfacing [189]. Choi and co-workers elegantly demonstrated how deimmunized functional antibody molecules can be created through structure-based design and simultaneous integration of HSC [147].

The generation of immune responses against a biotherapeutic requires multiple critical steps beyond reproducing human antibody sequence diversity [190]. Indeed, humanized and even fully human antibodies can elicit immune responses among the patients receiving such medicines and generate ADAs against them. Generation of ADAs is a multi-factorial issue and depends upon patients genetic background and disease history as well as quality attributes of the protein therapeutics, particularly, presence of aggregates and other degradants even in very minute quantities [190, 191]. A crucial first step towards ADA generation is the binding of short biotherapeutic-derived peptide fragments to major histocompatibility complex class II (MHC II) molecules. Several computational approaches have been developed to identify potential MHC I and MHC II binding T-cell epitopes as well as conformational B-cell epitopes [192]. Prediction of T-cell epitopes is addressed by machine learning approaches, particularly, neural networks-based methods that often rely on evaluating the binding affinity of a given short peptide towards MHC-I or II [192, 193]. In addition to such predictions, publicly available databases such as IEDB [18] provide free access to experimentally validated immunogenic peptide and protein sequences along with tools for their analyses. *In silico* predictions of potential MHC II binding T-cell immune epitopes in the amino acid sequences can be used as part of immunogenicity risk assessment and mitigation during the Lead Identification and Optimization stages along with other measures of humanness of the lead candidate sequences. In this regard, the immunogenicity scale developed by Epivax Inc. can be particularly useful [194] for initial triaging of the potential lead candidates and for formulating potential de-immunization or risk mitigation strategies. Kumar and co-workers have observed an overlap between potential immune epitopes and aggregation prone regions (APRs) around the CDRs of therapeutic antibodies [195, 196]. In addition to offering a potential mechanistic understanding of how protein aggregates can break immune tolerance, the existing overlaps between immune epitopes and APRs in CDRs of biotherapeutics open up exciting opportunities for simultaneous optimization of potency, solubility and safety of antibody-based biotherapeutics via rational structure-based design. Altogether, computational methods that facilitate deimmunization might offer a faster and cost-efficient way of pre-selecting molecules with better immunogenicity properties during the Lead Optimization stage. We must emphasize that connection between computational predictions of immune epitopes and ADAs generated against biotherapeutics remains largely untested. Therefore, it remains to be seen whether computational deimmunization strategies truly work in clinic.

### Biophysical properties

Together with immunogenicity, developing a working therapeutic also relies on favorable biophysical properties of the molecule. This includes properties such as colloidal stability of the antibody solution, concentration dependent viscosity behaviors and physicochemical degradation [197–201]. Good solubility is crucial [202, 203] to avoid aggregation that can potentially lead to loss of activity, degradation of antibodies or immunogenicity, as discussed previously. From a general perspective, protein aggregation remains a major unsolved problem in biochemistry. Aggregation has two aspects, namely, mechanistic and kinetic. Mechanistic aspects focus on protein instability and on identifying potential APRs, mainly hydrophobic patches on the protein surface, which can potentially nucleate aggregation. A number of groups have reviewed the applicability of various algorithms (Figure 2D, Table 2E) available to predict APRs in biotherapeutics [204, 205]. Wang and co-workers have examined molecular sequences of commercially available mAb drug products and shown that they contain multiple well defined aggregation prone motifs often located in their CDRs [206]. These CDR-located APRs also contribute significantly towards antigen binding [160], which help us rationalize how antibodies may lose potency upon aggregation and suggest potential strategies for selecting APRs for disruption without impacting biological activity. Recently, Rawat and co-workers have collected experimental data on aggregation kinetics available in literature and used machine learning to identify aggregation rate enhancer and mitigatory mutations in proteins [207]. Several generic predictors of solubility and APRs in proteins have been developed [208, 209] and though these have been successfully applied to antibodies [206], antibody-specific protocols addressing these issues also exist [204, 210]. Lauer and co/workers carried out a 2-year long measurement of biophysical properties for 12 antibodies [148] from which they derived a score, the developability index (DI), and demonstrated that it correlated well with the favorable biophysical properties of their antibodies. The DI combines the computed hydrophobicity, SAP score [211] and net charge of the molecule into a statistical score indicating APRs. Identification of hydrophobic regions is an important step in aggregation prediction that ideally requires a crystal structure of the antibody or a reliable homology model. This was addressed by Jain and colleagues who developed a surface accessible area predictor that can be applied to an antibody sequence to further create a propensity score that could be correlated with aggregation risk [149]. Measures such as the DI and the aggregation propensity risk score rely on the hydrophobicity scales and charge annotations, demonstrating that there is useful information in these parameters alone. An extended set of physico-chemical parameters was used by Obrezanova and colleagues [15] to create an Adaptive Boosting model for aggregation prediction. The model was trained and validated on a dataset of 500 antibodies with calculated biophysical properties.

The aforementioned methods relied on proprietary datasets of calculated aggregation propensity, data from clinical stage or marketed biotherapeutics in order to develop fast computational methods to perform pre-selection of candidates with more favorable developability properties during the Lead Optimization stage. An alternative approach is to use natural antibody sequences under the assumption that they have favorable biophysical properties [13]. In this study Raybould *et al.* stipulate five computational guidelines to define favorable biophysical properties in antibody therapeutics. Among these a structure-based hydrophobicity score is calculated and the value is then compared to the distribution of the same score in naturally sourced NGS sequences. Score values diverging significantly from the natural distribution are highlighted and the associated sequences flagged for developability risk. This work demonstrates a new paradigm in employing the vast amount of naturally-sourced NGS data to guide therapeutic antibody development.

## Developing trends

### Data-mining NGS

Development of computational methods to aid antibody engineering relies on successful exploration and exploitation of new data sources. In this respect, the field currently benefits from a steady stream of new data from NGS of B-cell Receptors (BCRs, to be used as proxy for antibodies) [212, 213] and there is an increasing number of resources available for downloading such data [17], which are being used to analyze therapeutic antibodies [13]. Current bioinformatic analyses of NGS repertoires focus on large-scale decoding of the immune responses, with several potential applications for therapeutic design [22, 25, 214].

One of the main applications of computational analysis of NGS outputs is the identification of antigen-specific BCR sequences after immunization (Figure 2E). Upon administering an immunogen to an organism, antigen-specific antibodies are raised, thus polarizing the immune repertoire. Sequencing a sample of the repertoire and identifying newly abundant sequence-similar BCRs is being used as a simple bioinformatic method to identify new antibodies specific for the antigen of interest. Clustering by V, J genes and CDRH3 sequence to identify large sequence-similar groups can identify antigen-specific cells after vaccinating humans with Hepatitis B [215]. A similar approach based on identifying highly abundant sequences was used to select antigen-specific molecules in immunized mice [216]. Such simple models however can still identify sequences that are not antigen-specific or miss less abundant antigen-specific sequences [215]. The selection of false positives can be tackled by more complex statistical models as highlighted by Fowler and co-workers [217]. Identification of antigen-specific antibodies after immunization can readily inform vaccine design since such antibodies can be used to confer passive immunity [218].

Identification of antigen-specific sequences can be naturally extended to detection of the immune state altogether (Figure 2E). Since the immune system is a dynamic reflection of the overall health of the organism, some antigen-specific signatures in the repertoire could be indicative of particular diseases [219]. It was demonstrated that statistical classifiers can identify immune profiles of patients with chronic lymphocytic leukemia [220], multiple sclerosis [221] or influenza [222] from NGS data alone. Further development of a larger variety of such models could result in versatile diagnostic tools for multiple conditions on the basis of an individual’s sequenced BCR repertoire [220].

Detection of antigen-specific sequences or the immune state could be improved by defining sequence- and structure-based rules governing adaptive immune responses through large scale analysis of antibody repertoires [25]. From a sequence perspective, it was demonstrated recently that despite the vast numbers of diverse sequences in a typical human antibody repertoire, a non-trivial amount of these is shared between individuals [23, 223]. Human antibody repertoires can maintain their fundamental sequence diversity despite the removal of as many as 50–90% of sequences [214]. Human antibody repertoires also appear to be constrained structurally, and this includes regions of the antibody that display great variability such as the CDRH3 loop [224]. Furthermore, many therapeutic CDRH3 loops can be found in naturally sourced NGS datasets, indicating a certain degree of convergence between antibodies raised experimentally and naturally occurring ones [225]. Studying such convergence of immune repertoires might reveal strategic preferences that have arisen through natural evolution. Such constraints can be readily adapted to library construction and used to identify binding antibodies (Figure 2E) [226]. This has already been demonstrated to some extent through analysis of antibodies derived from 600 donors [24] followed by construction of a library based on natural positional preferences [26]. Deriving binders from more naturally focused libraries might produce binders with more favorable biophysical and immunogenic properties.

At this stage, further development of bioinformatic methods for NGS analysis will depend as much on improving the algorithms as on the quality of the data itself. In addition, most of the NGS datasets produced to date do not offer paired H and L chain sequences. Further development of single-cell technology to provide paired NGS data [64, 227] will expand our ability to query the immune system by computational methods, paving the way for better antibody-based therapeutics.

### Alternative antibody formats—nanobodies

New approaches to develop antibody-based therapeutics are increasingly focused on different molecular formats. One of the most promising is the H chain only antibody, or nanobody that are naturally occurring in camelids (llamas, alpacas and camels) [27] and sharks [228, 229] (reviewed by Muyldermans [230] and Bannas *et al.* [27]). In line with its first therapeutic approval in 2018 (caplacizumab), increasing levels of interest in these molecules is demonstrated by recent nanobody-specific databases and analyses [57, 58, 231, 232].

The nanobody contains just three highly variable loops CDRH1, CDRH2 and CDRH3, which form an extended structural paratope located at one side of the folded protein domain. The absence of the L chain means that the nanobody’s CDRs are distinct from those of antibodies in both sequence and structure, and as a result nanobodies are able to bind antibody-inaccessible epitopes in enzyme active sites, viral capsids and G protein coupled receptors [233, 234]. Recent computational analyses of large sets of antibody and nanobody sequences and structures demonstrate that non-trivial systematic differences between these molecules exist [231, 232]. Specifically, nanobodies were found to exhibit less sequence and structure variation across their framework regions, and similar levels of sequence variation as classical antibodies within the CDRH1 and CDRH2 loops [232]. However, nanobodies translate this same level of sequence variation into increased structural diversity across the CDRH1 and CDRH2 loops, which are not classifiable by established canonical rules, presenting additional challenges to computational structural modelling tools compared to classical antibodies [232, 235]. Furthermore, the nanobody CDRH3 loop is on average three to four residues longer than its antibody counterpart, and significantly more diverse in terms of both primary sequence and tertiary structure configuration, defying the canonical antibody V_H_-domain-based rules and enabling nanobody CDRH3 loops to exhibit finger-like protrusions that extend into epitope cavities on their cognate antigens [230, 232, 235, 236].

Perhaps more significant in terms of challenges to computational modelling tools is that nanobody paratopes contain on average nearly three additional residues, and moreover the paratope is drawn from a wider set of aligned sequence positions than classical antibody V_H_ domain paratopes, comparable to the set used by the entire V_H_-V_L_ paratope [230, 232]. Given that the V_L_ domain is much less structurally variable, this suggests that the application of computational structural modelling tools to nanobody–antigen interactions will require innovation. In addition, analysis of large sets of nanobody–antigen co-crystal structures reveals that nanobody paratopes are made up of a much greater diversity of structural subunits, further increasing the modelling challenge [231]. CDRH3 loop residues that are highly variable in both sequence and structure dominate antigen-contacting residues within nanobodies, suggesting that the nanobody-antigen interface will be difficult to model using tools that have been developed in the context of classical antibody V_H_ domains. Because of such differences it is not clear whether the current methods for antibody modelling, docking etc. are directly transferable to nanobodies. Thus, a systematic benchmark of existing antibody computational tools would be highly informative to establish the extent to which these are applicable to nanobodies, and the innovations that are necessary to drive computational nanobody development.

## Conclusions

Antibodies continue to dominate the field of biotherapeutics with an increasing number of new clinical approvals each year. Current approaches to bring these molecules to the market have remained experimentally focused, with animal immunization and surface display technologies accounting for the majority of molecules developed to date. The increasing amount of antibody-specific data in the public domain facilitates the maturation of computational antibody design methods, resulting in a growing uptake as part of standard pharmaceutical discovery processes.

Computational methods are unlikely to replace the entire discovery process. Indeed, their largest added value will continue to be in providing time and cost-efficient ways of guiding experimental methods. Structural modelling can offer insight on exposed residues to be used for mutagenesis to either optimize binding, reduce immunogenicity or provide information on hydrophobicity patches related to detrimental biophysical properties. Predicting interface information can provide an initial guide for experimental epitope mapping efforts or offer a starting point for a therapeutic campaign by providing the basis for focused surface display libraries to design a novel antibody binder for a given epitope. Exploiting the vast amount of data generated by NGS will facilitate the derivation of more reliable ‘humanness’ and ‘developability’ profiles with which to guide antibody therapeutic discovery.

Existing computational antibody design knowledge and tools may benefit emerging biotherapeutic modalities akin to antibodies, such as nanobodies. However, despite the similarity between antibodies and nanobodies, systematic benchmarking will still be needed to determine whether development of nanobodies can benefit from computational antibody protocols in their current form or whether they need to be adjusted accordingly. Holistically, benchmarking of bioinformatic antibody methods on a par with existing protein-generic initiatives such as CASP, CAPRI or CAMEO [237] will benefit the entire computational antibody field. Antibody-specific benchmarking challenges will emphasize the shortcomings and advantages of each method and enable improvements to be developed in a focused manner, specifically with regard to their utility in therapeutic development process.

Further progress in the development of antibody-specific computational tools will be associated with access to more and diverse data in the public domain. It will become increasingly important that these data adhere to information management and reusability best practices. Such efforts are exemplified by AIRR community, which aims to standardize the increasing amount of antibody NGS depositions and their metadata [213], and from a broader perspective by the adoption of scientific data management principles such as FAIR [238]. Organizations involved in the discovery and development of antibody therapeutics have a unique opportunity to catalyze the development of the computational antibody methods by participating in data sharing and benchmarking efforts. Publishing proprietary data, which has no or little commercial value, generated in the process of developing a candidate therapeutic may yield a higher return in the form of better computational methods.

As the importance of antibodies as therapeutics grows, faster and more accurate computational methods are set to become even more tightly integrated into therapeutic development processes, thus accelerating the delivery of new medicines to patients.

**Key Points**

- Antibodies are the largest group of biopharmaceuticals.
- Established bioinformatics methods such as protein structural homology modelling and molecular docking can be readily applied to the specific case of antibodies in a therapeutic setting.
- Increasing amount of data from next generation sequencing holds the potential to improve the computational methods and thus its applicability to therapeutic design.
- Computational antibody methods might be transferable to other immunoglobulin formats such as nanobodies, which have more potential in certain therapeutic areas.
- Systematic benchmarking of the tools can emphasize the advantages of computational methods and where these can be used to support therapeutic pipelines.

## Supplementary Material

Supplementary_Information_bbz095

## Notes

**Richard A. Norman** is a Consultant at Pistoia Alliance Inc., USA, and Managing Director at Norman Consulting, Norway. His interests lie in structural and computational biology, therapeutic discovery and innovation in the life sciences.

## Notes

Francesco Ambrosetti is a PhD candidate at Utrecht University. He is interested in integrative modeling of antibody–antigen complexes.

## Notes

**Alexandre M.J.J. Bonvin** is professor of Computational Structural Biology at Utrecht University in the Netherlands. His group is developing the integrative modelling platform HADDOCK and associated computational services.

## Notes

**Lucy J. Colwell** is a lecturer in Chemistry at the University of Cambridge. Her group studies the relationship between protein sequence and structure/function.

## Notes

**Sebastian Kelm** is an employee of UCB Celltech. He is interested in the three-dimensional modelling of protein structures and interactions with a view towards designing medicines that impact patients’ lives.

## Notes

**Sandeep Kumar** works for Boehringer Ingelheim. He is advocating for Biopharmaceutical Informatics, a strategic combination of Bioinformatics, Computational Biophysics and Computer Science to enable discovery and development of biologic medicines.

## Notes

**Konrad Krawczyk** works for NaturalAntibody. He is interested in computational techniques to streamline discovery of antibody-based biotherapeutics.
