# Supplementary material for: Computational approaches to therapeutic antibody design: established methods and emerging trends
Source: Brief Bioinform. 2019 Oct 18;21(5):1549–67. doi: 10.1093/bib/bbz095 (PMC7947987; doi:10.1093/bib/bbz095)
Supplement: Supplementary_Information_bbz095 [file supplementary_information_bbz095.docx]

**Supplementary Information**

Computational approaches to therapeutic antibody design: established methods and emerging trends.

Richard A. Norman, Francesco Ambrosetti, Alexandre M.J.J. Bonvin, Lucy J. Colwell, Sebastian Kelm, Sandeep Kumar and Konrad Krawczyk

**Supplementary Section 1. Database content descriptions.**

To facilitate understanding of the available data sources we provide a brief description of contents for each database in Table 1 and, wherever possible, a small number of example entries. Extensive search functionalities and Copyrighting prevented us from providing example entries for all databases.

**TABS – Therapeutic Antibody Database**

Accessible at: https://tabs.craic.com/users/sign_in

TABS is a commercial database containing information on therapeutic antibodies gathered from public sources. Users can identify therapeutic antibodies, their targets, parent companies and sequences.

**Therapeutic Antibody Database in SAbDab (TAbDab)**

Accessible at: http://opig.stats.ox.ac.uk/webapps/sabdab-sabpred/Therapeutic.html

The TAbDab website offers structural mapping between monoclonal antibody therapeutics and structures in the Protein Data Bank. Exact and near matches to the therapeutic variable domain sequence are provided (Supplementary Figure 1).


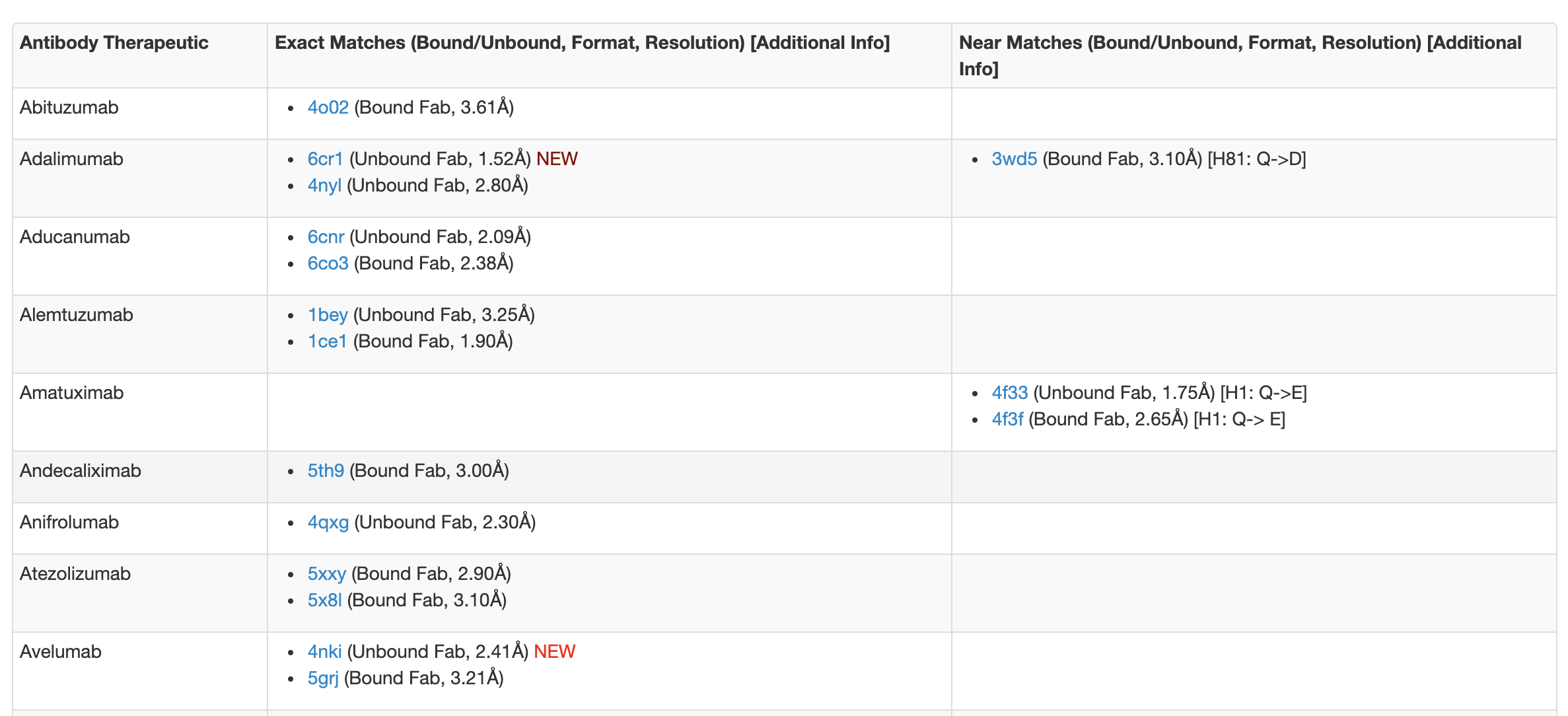


**Supplementary Figure 1.** Sample contents for TAbDab.

**Andrew Martin’s antibody pages.**

Accessible at: <http://www.bioinf.org.uk/abs/>

Andrew Martin’s antibody resources page at UCL offers links to antibody related tools developed at UCL. It also provides an overview of antibody structure and sequences as well as information on antibody numbering.

**AAAAA – Aho’s Amazing Atlas of Antibody Anatomy.**

Accessible at: <https://www.bioc.uzh.ch/plueckthun/antibody/index.html>

AAAAA is a website offering resources on antibody structure and function. Readers can compare the different numbering schemes as well as download structural and sequence datasets relating to antibodies collected by the authors.

**AbMiner**

Accessible at: <https://discover.nci.nih.gov/abminer/>

AbMiner is a search service on available monoclonal antibodies. Epitope information is given where available together with the immunogen (Supplementary Figure 2). The contents of the database can be retrieved by specifying one of a variety of filters such as antibody name, gene or molecular weight.


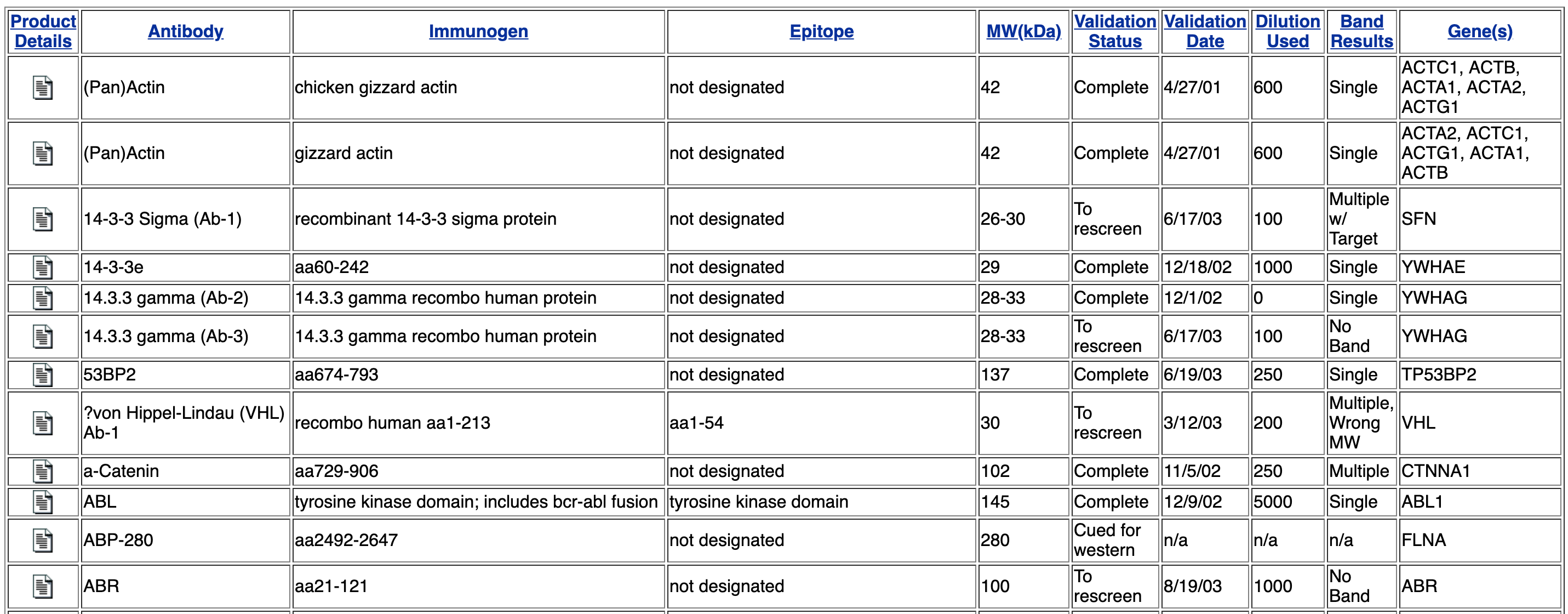


**Supplementary Figure 2.** Sample contents of AbMiner.

**IgPdb**

Accessible at: http://cgi.cse.unsw.edu.au/~ihmmune/IgPdb/information.php

This database encompasses known alternative allelic variants of antibodies (Supplementary Figure 3). The variants can be searched by specifying the IMGT gene name or by publication keywords. The information in the database provides an alternative to the allelic variants curated by IMGT.


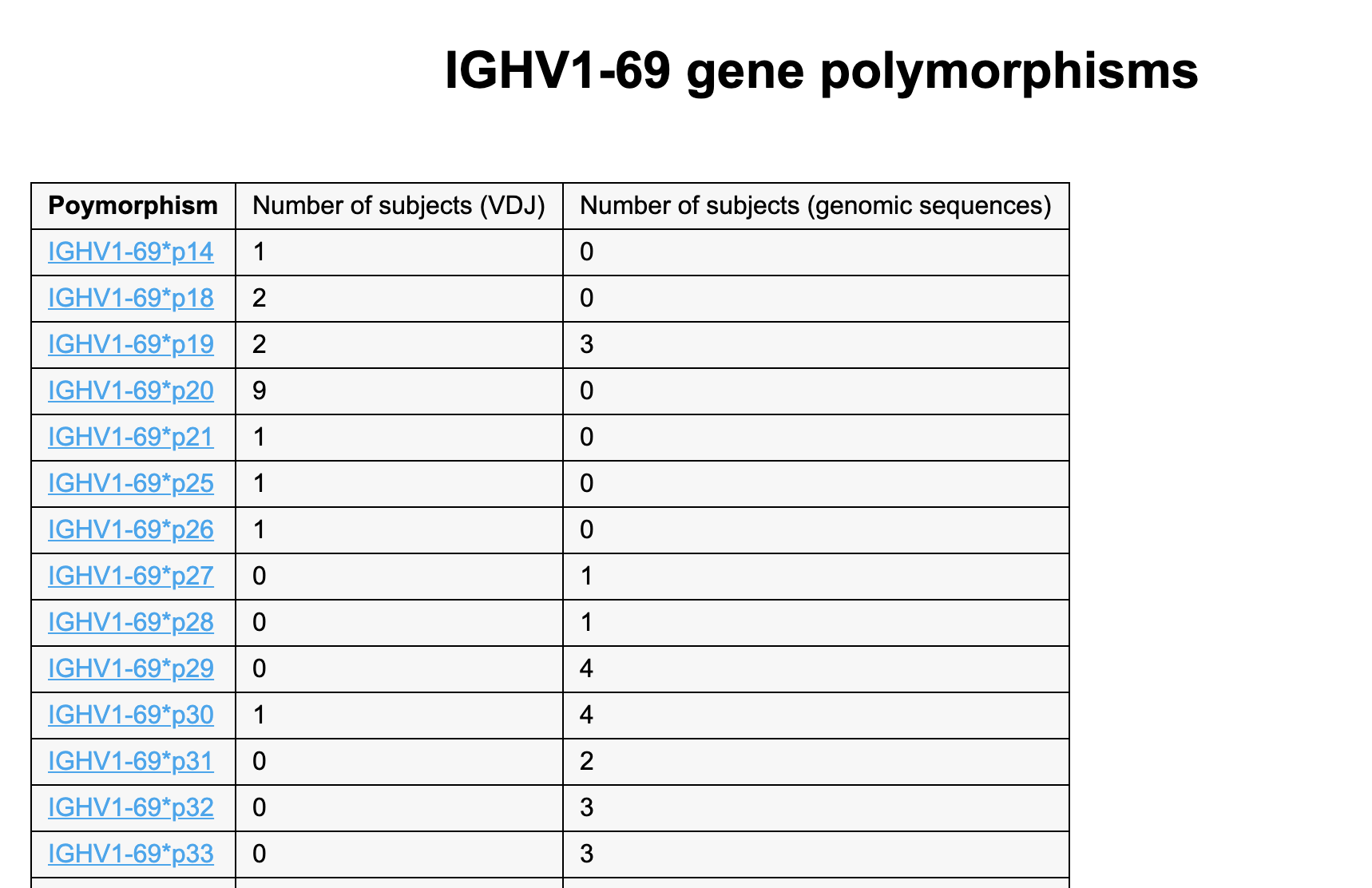


**Supplementary Figure 3.** Example contents of IgPdb.

**IMGT**

Accessible at: http://www.imgt.org/

IMGT offers databases of antibody genes, structures and sequences. It provides services to perform annotation of raw antibody data and a reference for their own antibody numbering scheme. IMGT is currently the main reference for antibody germlines.

**Abysis**

Accessible at: http://www.abysis.org/

Abysis is a database of antibody sequences and structures with associated analysis tools. Users can perform searches for sequences and structures using a variety of filters such as source organism and antigen type.

**DIGIT**

Accessible at: http://circe.med.uniroma1.it/digit/help.php

DIGIT is a database of antibody sequences collected from NCBI single sequence depositions. Users can perform searches by providing source organism and antigen type or by using the variable region sequence of interest to identify close sequence identity matches in the database. The results show the descriptions of the antibodies with their literature references (Supplementary Figure 4).


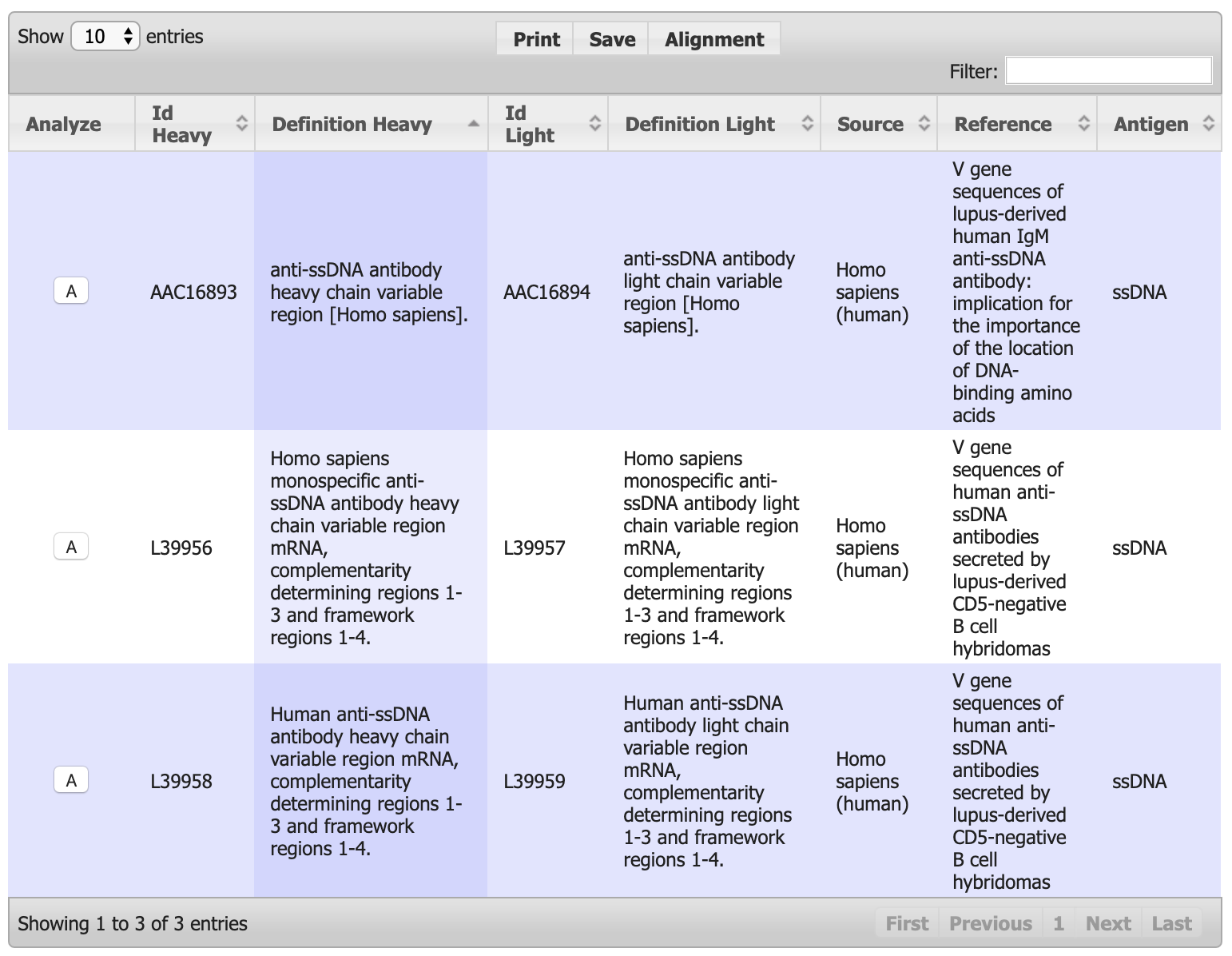


**Supplementary Figure 4.** Sample contents of DIGIT.

**iReceptor**

Accessible at: http://ireceptor.irmacs.sfu.ca/

iReceptor curates antibody data from raw Next Generation Sequence reads. The raw sequence reads are annotated with antibody-specific information such as CDRs and are made available for retrieval.

**Observed Antibody Space**

Accessible at: <http://antibodymap.org/oas>

This database curates antibody data from Next Generation Sequence reads (Supplementary Figure 6). The data are annotated with antibody-specific information (e.g. CDRs, IMGT-numbering) and are linked to the experimental data used to derive the sequences (immune state, sequencing type). A search on metadata entries is available allowing the users to bulk download subsets of the database.


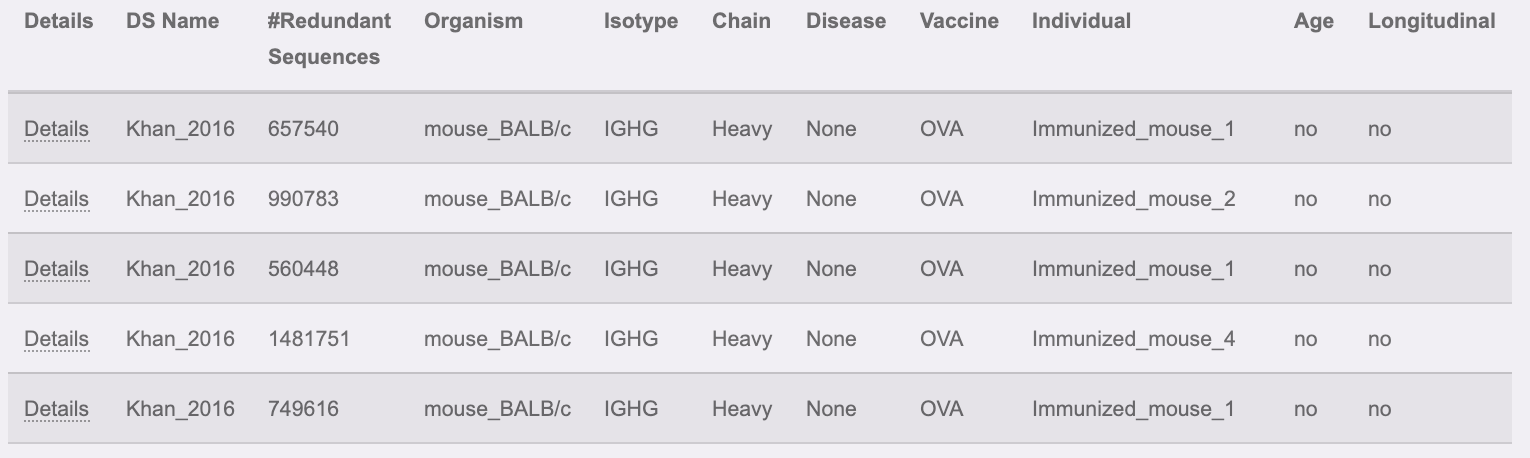


**Supplementary Figure 6.** Sample contents of OAS.

**SystimsDB**

Accessible at: [https://www.systimsdb.ethz.ch](https://www.systimsdb.ethz.ch/)

SystimsDB contains antibody sequences from Next Generation Sequencing experiments. The data are available for retrieval using a wide variety of filters such as CDR sequence and antigen type.

**PCLICK**

Accessible at: http://mspc.bii.a-star.edu.sg/minhn/cluster_pclick.html

PCLICK is a database of antibody-antigen complexes that share binding site similarities. The database is aimed at providing common binding motifs in antibody-antigen recognition. The data are available as a bulk download.

**PyIGClassify**

Accessible at: http://dunbrack2.fccc.edu/PyIgClassify/

PyIGClassify offers CDR canonical class annotations for structures in the PDB. Users can input a PDB code to provide the canonical class annotations for the given structure (Supplementary Figure 6).


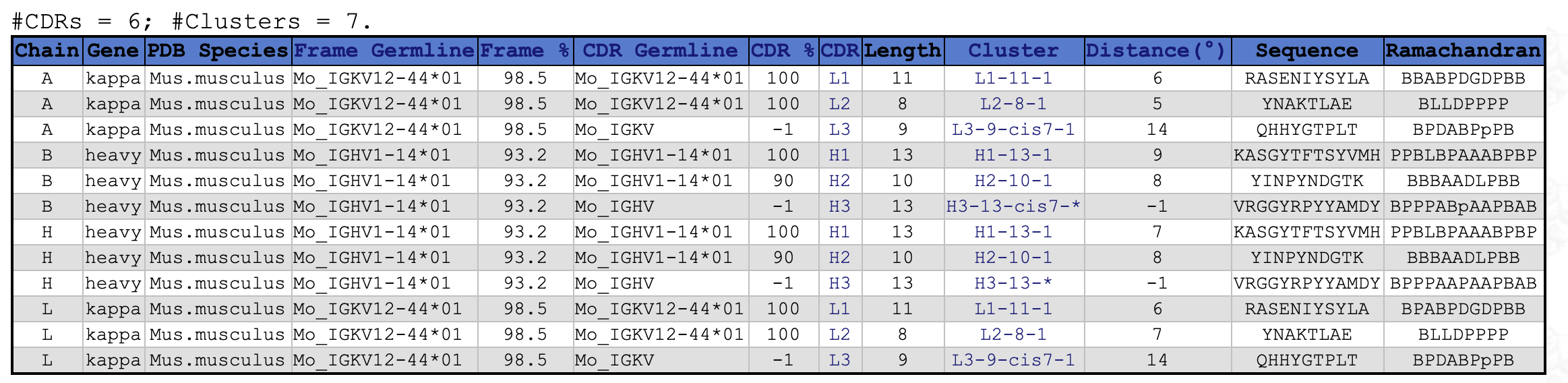


**Supplementary Figure 6.** Sample contents of PyIgClassify.

**SAbDab**

Accessible at: http://opig.stats.ox.ac.uk/webapps/sabdab-sabpred/Welcome.php

The Structural Antibody Database is a collection of antibody structures found in the PDB. Users can perform searches for structures using diverse filters such as RMSD, organism or presence of an antigen (Supplementary Figure 7). The database updates itself on a weekly basis, synchronous with the PDB.


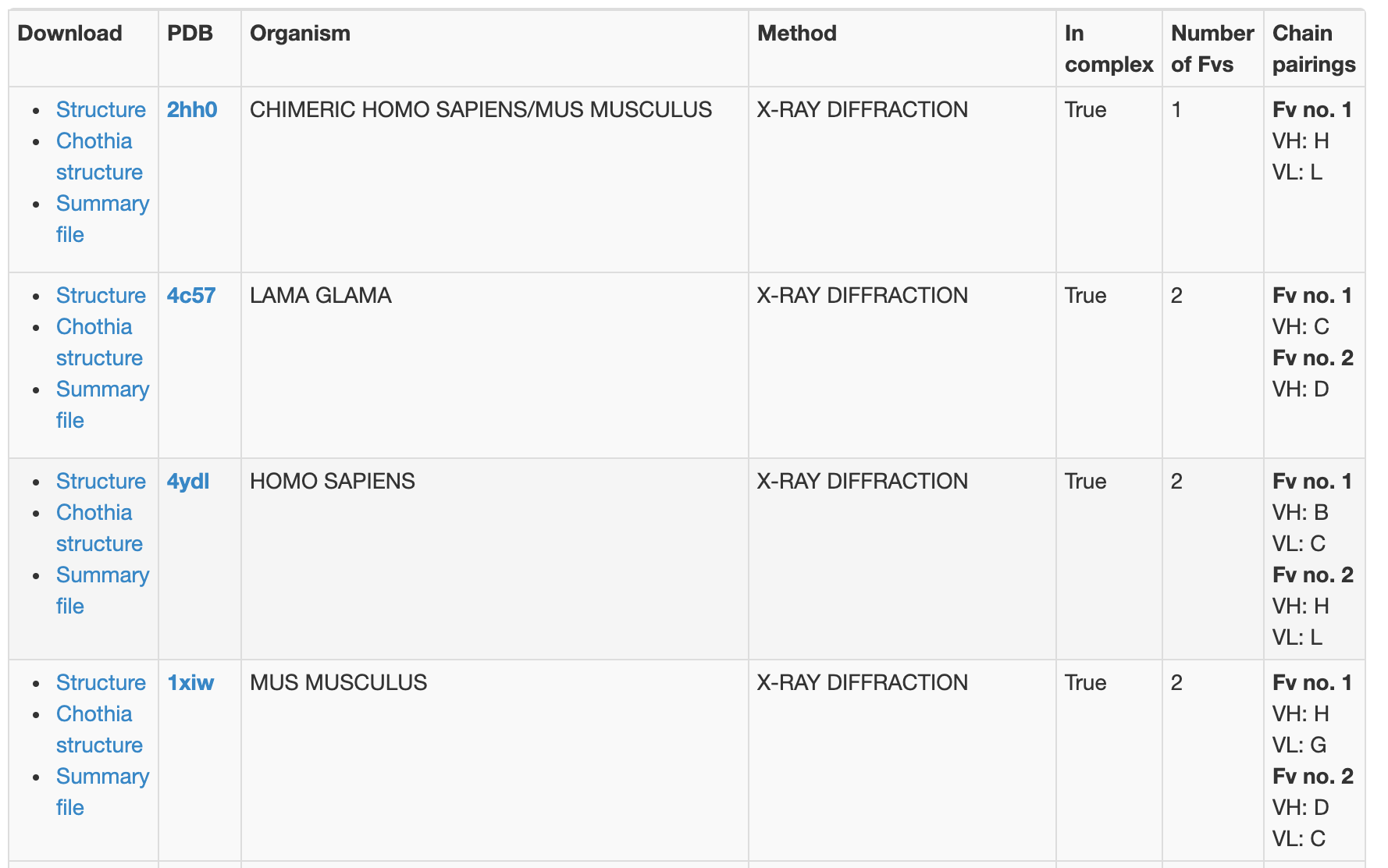


**Supplementary Figure 7.** Sample contents of SAbDab.

**ABDB**

Accessible at: http://www.bioinf.org.uk/abs/abdb/

ABDB is database of antibody structures from the PDB (Supplementary Figure 8). Users can retrieve the structures of particular antibodies using a variety of filters such as the PDB code, antibody name or organism. The data are also available for bulk download.


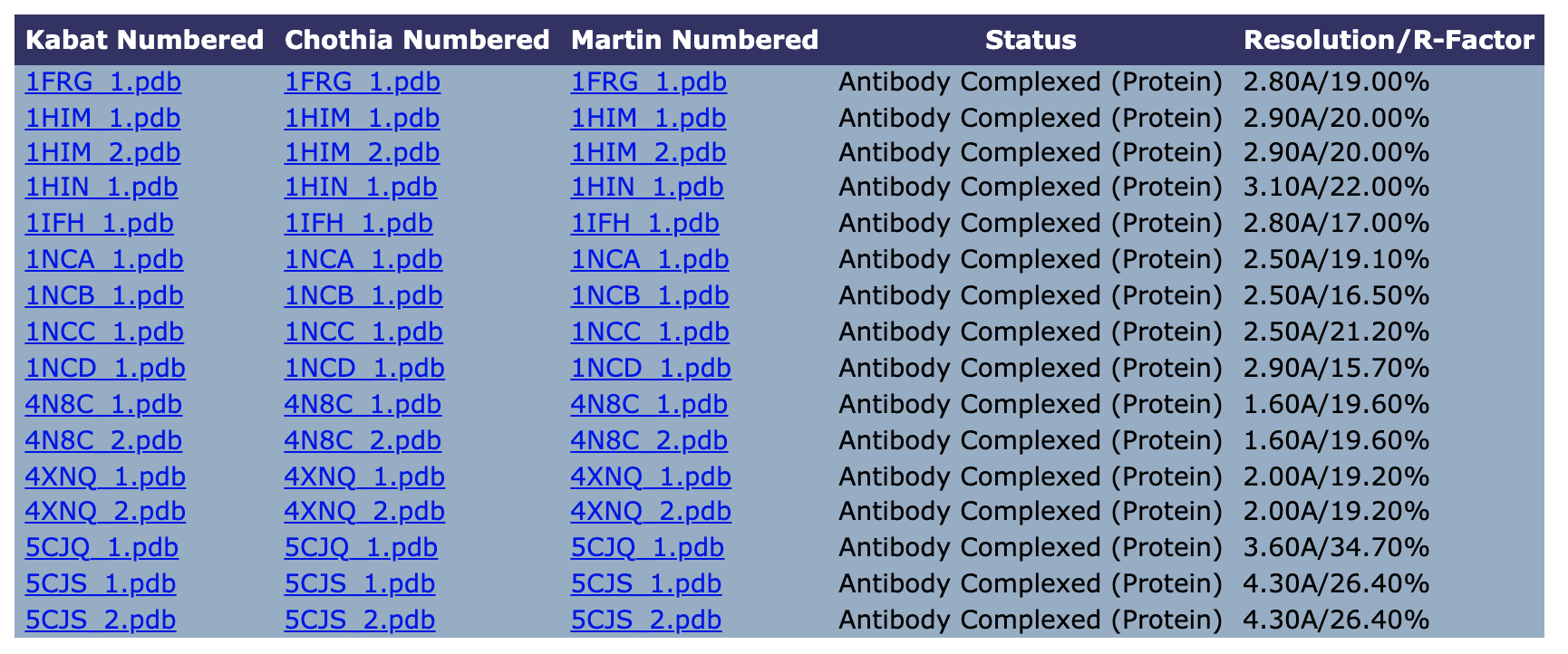


**Supplementary Figure 8.** Sample contents of ABDB.

**IEDB**

Accessible at: http://iedb.org

IEDB is a collection of epitopes collected from publicly available sources such as the PDB (Supplementary Figure 9). IEDB curates the antibody sequences linked to the specific epitopes and users can search the database using a variety of filters such as organism or antigen name. The database also offers an analysis resource providing tools for epitope prediction.


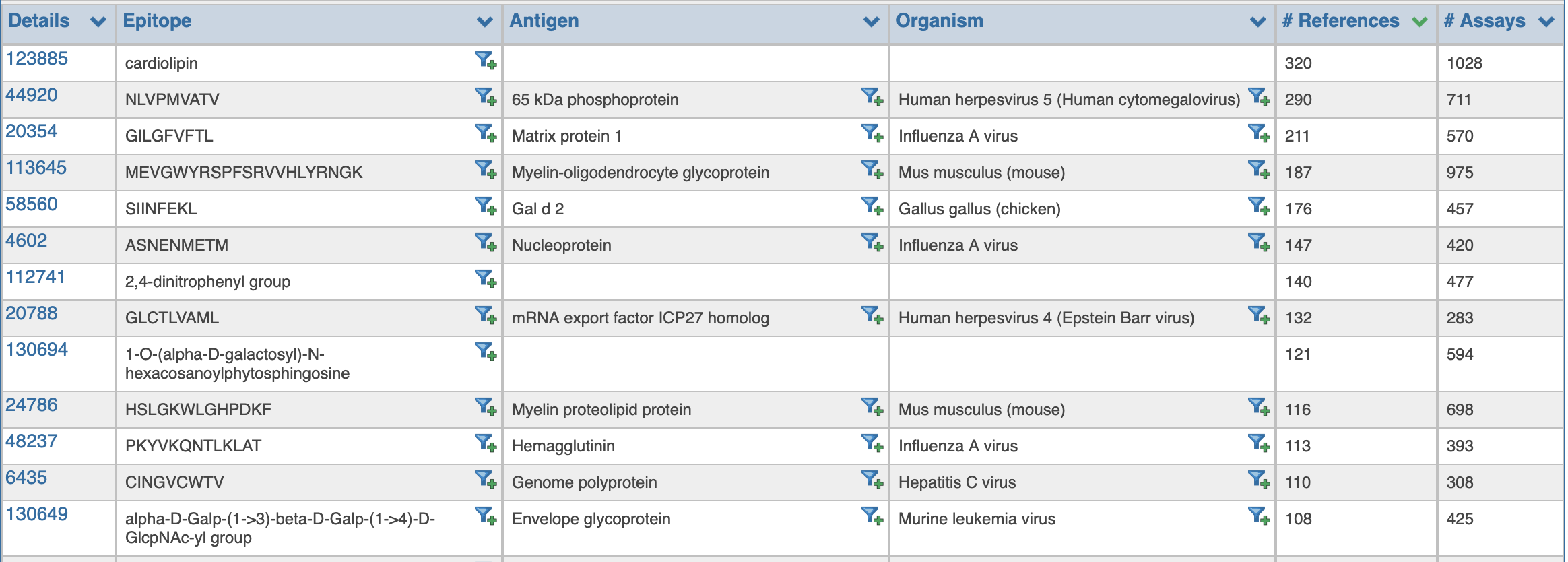


**Supplementary Figure 9.** Sample contents of IEDB.

**AntigenDB**

Accessible at: <http://crdd.osdd.net/raghava/antigendb/>

AntigenDB curates information on validated antigens (Supplementary Figure 10). Users conduct sequence-based searches to identify antigens and their corresponding epitopes together with various annotations such as source organism, structure model and surface access.


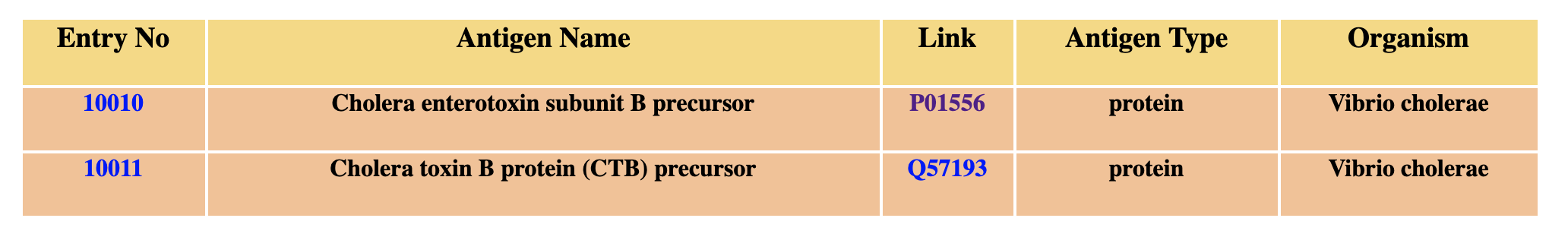


**Supplementary Figure 10.** Sample contents of AntigenDB.

**PDBBind**

Accessible at: http://www.pdbbind.org.cn/

PDBBind collects the data on structures from the PDB with reported affinity information. Antibodies make up a subset of this database. Upon registration the entire dataset can be downloaded which offers PDB codes together with experimentally measured binding affinity data (Kd, Ki, IC50).

**Ab-Bind**

Accessible at: <https://github.com/sarahsirin/AB-Bind-Database>

Ab-Bind was created by performing mutations to antibody-antigen complexes in the PDB and measuring their binding affinities and is a useful reference for developers of binding affinity predictors.

**SKEMPI**

Accessible at: https://life.bsc.es/pid/skempi2/

SKEMPI is a database of PDB complexes and their mutants with associated energetic binding affinity information (Supplementary Figure 11). The database encompasses proteins in general, of which antibodies are a subset. It is useful for developers of binding affinity prediction methods.


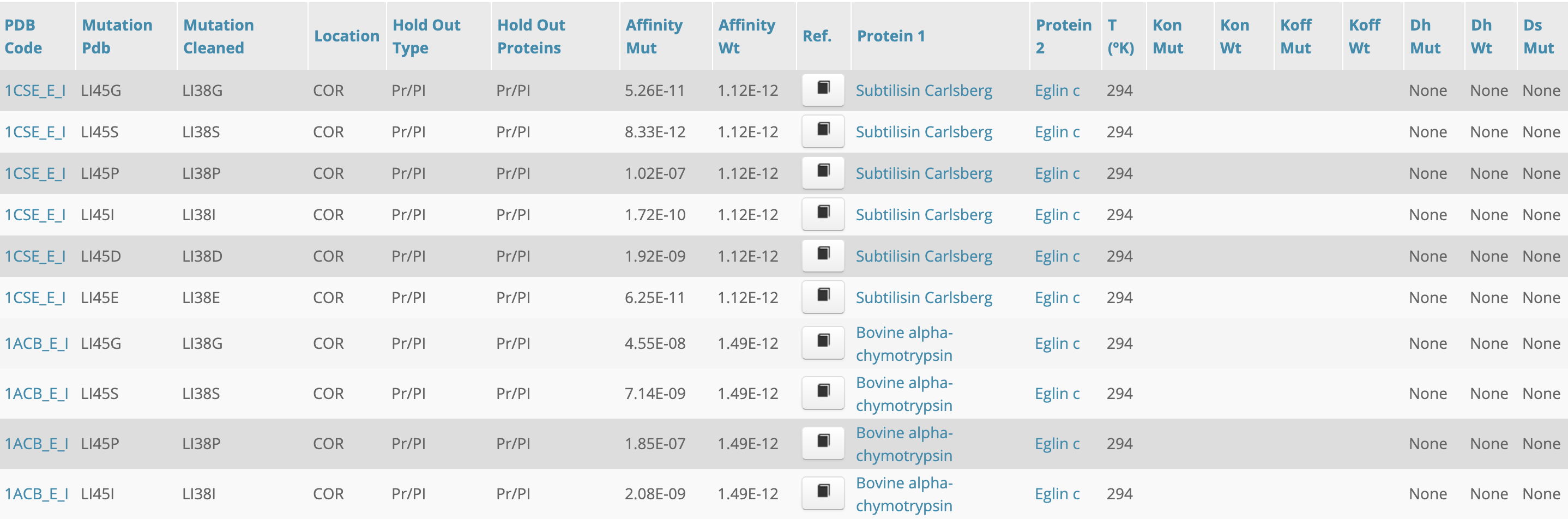


**Supplementary Figure 11.** Sample contents of SKEMPI.

**Non-redundant Nanobody database.**

Accessible from the publication: https://www.sciencedirect.com/science/article/pii/S2352340919301052

Provides a non-redundant list of sequences for which nanobody structures exist. This database should facilitate structural studies of nanobodies.

**SDAb-DB**

Accessible at: http://sdab-db.ca/

The database of single domain antibodies contains sequences and structures of nanobodies in the public domain. The nanobodies can be searched using variety of filters, such as nanobody name, source organism or the amino acid sequence (Supplementary Figure 12). The result of the query are links to antibodies matching the filters.


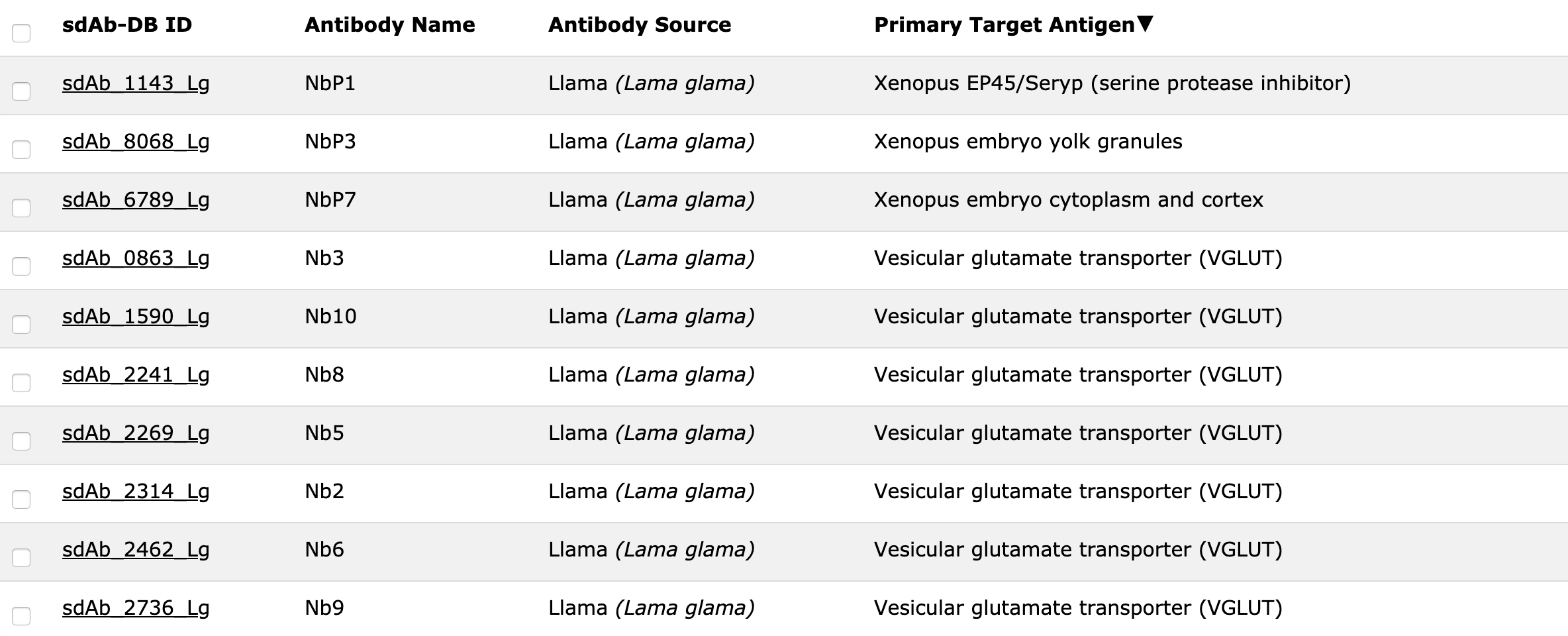


**Supplementary Figure 12.** Sample contents of SDAb-DB.

**iCAN**

Accessible at: http://ican.ils.seu.edu.cn/

The iCAN database curates nanobody information from the public domain, including patents, to advance general research and clinical application of nanobodies. Nanobodies can be retrieved via sequence search or by using a variety of filters such as organism or targeted antigen.

Supplementary Table 1. Linear Epitope Predictors.

| **Method Name** | **Role** | **Link** | **Reference** |
| --- | --- | --- | --- |
| LBTope | Linear Epitope Prediction | <http://crdd.osdd.net/raghava/lbtope/> | [1] |
| SVMTriP | Linear Epitope Prediction | <http://sysbio.unl.edu/SVMTriP/> | [2] |
| ABCpred | Linear Epitope Prediction | <http://www.imtech.res.in/raghava/abcpred/> | [3] |
| iBCE-EL | Linear Epitope Prediction | <http://thegleelab.org/iBCE-EL/> | [4] |
| BcePred | Linear Epitope Prediction | <http://www.imtech.res.in/raghava/bcepred/> | [5] |
| Bepipred 2 | Linear Epitope Prediction | - | [6] |

Supplementary Table 2. Conformational Epitope Predictors.

| **Method Name** | **Role** | **Link** | **Reference** |
| --- | --- | --- | --- |
| CBTOPE | Conformational Epitope Prediction | <http://www.imtech.res.in/raghava/cbtope/> | [7] |
| BETOP | Conformational Epitope Prediction | - | [8] |
| CEP | Conformational Epitope Prediction | - | [9] |
| 3DEX | Conformational Epitope Prediction | - | [10] |
| DISCOTOPE | Conformational Epitope Prediction | <http://www.cbs.dtu.dk/services/DiscoTope/> | [11] |
| EpiSearch | Conformational Epitope Prediction | <http://curie.utmb.edu/episearch.html> | [12] |
| Ellipro | Conformational Epitope Prediction | <http://tools.iedb.org/ellipro/> | [13] |
| GLEP | Conformational Epitope Prediction | http://github.com/lzhlab/glep/ | [14] |
| Bepro (PEPITO) | Conformational Epitope Prediction | <http://pepito.proteomics.ics.uci.edu/> | [15] |
| LocaPep | Conformational Epitope Prediction | - | [16] |
| SEPPA | Conformational Epitope Prediction | <http://badd.tongji.edu.cn/seppa/> | [17,18] |
| SEPIa | Conformational Epitope Prediction | <https://github.com/gdalkas/SEPIa> | [19] |
| EPSVR | Conformational Epitope Prediction | http://sysbio.unl.edu/EPSVR/ | [20] |
| Epitopia | Conformational Epitope Prediction | http://epitopia.tau.ac.il/ | [21] |
| EPCES | Conformational Epitope Prediction | <http://sysbio.unl.edu/EPCES/> | [22] |

**References**

1. Singh H, Ansari HR, Raghava GPS. Improved Method for Linear B-Cell Epitope Prediction Using Antigen’s Primary Sequence. PLoS One 2013;

2. Yao B, Zhang L, Liang S, et al. SVMTriP: A Method to Predict Antigenic Epitopes Using Support Vector Machine to Integrate Tri-Peptide Similarity and Propensity. PLoS One 2012;

3. Saha S, Raghava GPS. Prediction of continuous B-cell epitopes in an antigen using recurrent neural network. Proteins Struct. Funct. Genet. 2006;

4. Manavalan B, Govindaraj RG, Shin TH, et al. iBCE-EL: A New Ensemble Learning Framework for Improved Linear B-Cell Epitope Prediction. Front. Immunol. 2018;

5. Saha S, Raghava GPS. BcePred: Prediction of Continuous B-Cell Epitopes in Antigenic Sequences Using Physico-chemical Properties. 2010;

6. Jespersen MC, Peters B, Nielsen M, et al. BepiPred-2.0: Improving sequence-based B-cell epitope prediction using conformational epitopes. Nucleic Acids Res. 2017;

7. Ansari HR, Raghava GP. Identification of conformational B-cell Epitopes in an antigen from its primary sequence. Immunome Res. 2010;

8. Zhao L, Wong L, Lu L, et al. B-cell epitope prediction through a graph model. BMC Bioinformatics 2012;

9. Kulkarni-Kale U, Bhosle S, Kolaskar AS. CEP: A conformational epitope prediction server. Nucleic Acids Res. 2005; 33:

10. Schreiber A, Humbert M, Benz A, et al. 3D-Epitope-Explorer (3DEX): Localization of conformational epitopes within three-dimensional structures of proteins. J. Comput. Chem. 2005;

11. Haste Andersen P, Nielsen M, Lund O. Prediction of residues in discontinuous B-cell epitopes using protein 3D structures. Protein Sci. 2006; 15:2558–2567

12. Negi SS, Braun W. Automated detection of conformational epitopes using phage display peptide sequences. Bioinform. Biol. Insights 2009;

13. Ponomarenko J, Bui H-H, Li W, et al. ElliPro: a new structure-based tool for the prediction of antibody epitopes. BMC Bioinformatics 2008; 9:514

14. Zhao L, Wu S, Jiang J, et al. Novel overlapping subgraph clustering for the detection of antigen epitopes. Bioinformatics 2018;

15. Sweredoski MJ, Baldi P. PEPITO: Improved discontinuous B-cell epitope prediction using multiple distance thresholds and half sphere exposure. Bioinformatics 2008; 24:1459–1460

16. Pacios LF, Tordesillas L, Palacín A, et al. LocaPep: Localization of epitopes on protein surfaces using peptides from phage display libraries. J. Chem. Inf. Model. 2011;

17. Qi T, Qiu T, Zhang Q, et al. SEPPA 2.0 - More refined server to predict spatial epitope considering species of immune host and subcellular localization of protein antigen. Nucleic Acids Res. 2014; 42:

18. Sun J, Wu D, Xu T, et al. SEPPA: A computational server for spatial epitope prediction of protein antigens. Nucleic Acids Res. 2009; 37:

19. Dalkas GA, Rooman M. SEPIa, a knowledge-driven algorithm for predicting conformational B-cell epitopes from the amino acid sequence. BMC Bioinformatics 2017;

20. Liang S, Zheng D, Standley DM, et al. EPSVR and EPMeta: prediction of antigenic epitopes using support vector regression and multiple server results. BMC Bioinformatics 2010; 11:381

21. Rubinstein ND, Mayrose I, Martz E, et al. Epitopia: a web-server for predicting B-cell epitopes. BMC Bioinformatics 2009; 10:287

22. Liang S, Zheng D, Zhang C, et al. Prediction of antigenic epitopes on protein surfaces by consensus scoring. BMC Bioinformatics 2009; 10:302
